# Extra‐large G‐proteins influence plant response to *Sclerotinia sclerotiorum* by regulating glucosinolate metabolism in *Brassica juncea*

**DOI:** 10.1111/mpp.13096

**Published:** 2021-08-10

**Authors:** Ruchi Tiwari, Jagreet Kaur, Naveen C. Bisht

**Affiliations:** ^1^ National Institute of Plant Genome Research New Delhi India; ^2^ Department of Genetics University of Delhi South Campus New Delhi India

**Keywords:** *Brassica juncea*, extra‐large G‐proteins (XLG), glucosinolates, plant defence, *Sclerotinia sclerotiorum*

## Abstract

Heterotrimeric G‐proteins are one of the highly conserved signal transducers across phyla. Despite the obvious importance of G‐proteins in controlling various plant growth and environmental responses, there is no information describing the regulatory complexity of G‐protein networks during pathogen response in a polyploid crop. Here, we investigated the role of extra‐large G‐proteins (XLGs) in the oilseed crop *Brassica juncea*, which has inherent susceptibility to the necrotrophic fungal pathogen *Sclerotinia* *sclerotiorum*. The allotetraploid *B*. *juncea* genome contains multiple homologs of three *XLG* genes (two *BjuXLG1*, five *BjuXLG2*, and three *BjuXLG3*), sharing a high level of sequence identity, gene structure organization, and phylogenetic relationship with the progenitors’ orthologs. Quantitative reverse transcription PCR analysis revealed that *BjuXLGs* have retained distinct expression patterns across plant developmental stages and on *S*. *sclerotiorum* infection. To determine the role of *BjuXLG* genes in the *B*. *juncea* defence response against *S*. *sclerotiorum*, RNAi‐based suppression was performed. Disease progression analysis showed more rapid lesion expansion and fungal accumulation in BjuXLG‐RNAi lines compared to the vector control plants, wherein suppression of *BjuXLG3* homologs displayed more compromised defence response at the later time point. Knocking down *BjuXLGs* caused impairment of the host resistance mechanism to *S*. *sclerotiorum,* as indicated by reduced expression of defence marker genes *PDF1.2* and *WRKY33* on pathogen infection. Furthermore, BjuXLG‐RNAi lines showed reduced accumulation of leaf glucosinolates on *S*. *sclerotiorum* infection, wherein aliphatic glucosinolates were significantly compromised. Overall, our data suggest that *B*. *juncea* *XLG* genes are important signalling nodes modulating the host defence pathways in response to this necrotrophic pathogen.

## Graphical abstract

The *Brassica juncea XLG* genes play distinct roles in defence against *Sclerotinia sclerotiorum* through altered expression of defence pathway genes and leaf glucosinolates accumulation.

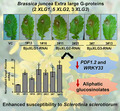

## 1 INTRODUCTION

Signal transduction is one of the important biological processes through which organisms perceive extracellular stimuli and transmit it intracellularly. Heterotrimeric G‐proteins (G‐proteins), consisting of G‐alpha (Gα), G‐beta (Gβ), and G‐gamma (Gγ) subunits, are a class of signal transducers conserved across eukaryotes. The model plant species *Arabidopsis thaliana* has a limited set of core G‐protein subunits, with a canonical Gα and Gβ, together with three Gγ subunits (Urano et al., 2013). The *Arabidopsis* genome additionally encodes three extra‐large Gα‐like proteins (XLGs), namely, XLG1, XLG2, and XLG3 (Ding et al., 2008; Lee & Assmann, 1999). XLGs are twice as large as the canonical Gα protein, which led to their terminology as extra‐large GTP binding proteins. The C‐terminal region of XLGs shows structural homology to canonical Gα proteins; however, their N‐terminal region is quite distinct (Ding et al., 2008). Despite the differences, XLGs show physical interaction with all three possible Gβγ dimers at the plasma membrane (Chakravorty et al., 2015; Heo et al., 2012; Maruta et al., 2015). XLGs thus represent additional nodes in plant G‐protein signalling, having multiple roles during plant development and stress responses (Ding et al., 2008; Maruta et al., 2015; Pandey, 2019; Urano et al., 2016).

Various studies using inhibitors, agonists, and loss‐of‐function mutants suggest the role of G‐protein signalling in plant defence (Trusov et al., 2009; Urano et al., 2016; Zhong et al., 2019). In general, a higher susceptibility response to bacterial and fungal pathogens was observed in the *Arabidopsis* Gβ‐null mutant (*agb1*), *xlg123*‐triple mutant, and Gγ triple mutant (*agg123*), but not in the null mutant of the canonical Gα (*gpa1*) and Col‐0 wild type, indicating that biotic stress responses are biased towards XLGs rather than canonical Gα (Urano et al., 2016). The expression of *XLG2* and *XLG3* genes was found to be rapidly induced by the bacterial pathogen *Pseudomonas syringae*, whereas *XLG1* transcription was not affected (Ding et al., 2008). Studies in *Arabidopsis* demonstrated that XLGs are positive regulators of resistance to the biotrophic pathogen *P*. *syringae*, which largely triggers the salicylic acid (SA)‐responsive pathway; however, no significant difference was observed for the necrotrophic fungi *Alternaria brassicicola* and *Botrytis cinerea*, which trigger the jasmonic acid (JA)‐responsive pathway (Maruta et al., 2015; Zhu et al., 2009). The *xlg2* mutation leads to compromised induction of pathogen‐responsive (*PR1* and *PR2*) genes during infected conditions. In addition, XLG‐mediated resistance against hemibiotrophic fungi and bacteria is associated with receptor‐like kinases (RLKs) and the defence mechanism is based upon the activation of programmed cell death (PCD). The *Arabidopsis* XLGs thus have distinct functions in disease resistance through their interaction with the Gβγ dimer (Zhong et al., 2019).

The genus *Brassica* is economically the most important genus of the Cruciferae family, crops of which are used for human nutrition as vegetables, oilseeds, and condiments. The inherent susceptibility of *Brassica* species to biotic stresses is a major factor that has limited their productivity in recent decades. *Sclerotinia sclerotiorum,* the causal agent of sclerotinia stem rot in *Brassica* crops, causes extensive (40%–80%) yield losses worldwide (Sharma et al., 2018) and has proved hard to control, with host resistance being insufficient. The persistence of this fungus is due to its sclerotia, which remain viable under adverse conditions and can be retained in soil for many years (Brustolin et al., 2016). This aggressive fungus is known to hijack plant defence by modulating a wide range of signalling cascades, defence phytohormones, and stress‐associated metabolites (Liang & Rollins, 2018; Novakova et al., 2014). Recent reports in *A*. *thaliana* and *Brassica* species have established the key role of glucosinolates and their hydrolysis products against the necrotrophic pathogen *S*. *sclerotiorum* (Abuyusuf et al., 2018; Augustine & Bisht, 2015a; Chen et al., 2020, 2021; Hopkins et al., 2009; Sotelo et al., 2015).

*Brassica* species have polyploid genomes, serving as an excellent crop model to study genome evolution and trait domestication (Augustine et al., 2014). We earlier reported that the mesohexaploid genomes of *Brassica rapa* and *Brassica nigra* contain multiple homologs of G‐protein genes, which show distinct expression patterns in response to defence phytohormones and conditions mimicking biotic stress, suggesting putative involvement of G‐protein signalling in plant defence (Arya et al., 2014; Kumar et al., 2014). There is a growing consensus that the signalling molecules, particularly JA, SA, and ethylene (ET), interact in complex ways to fine‐tune plant defence metabolites, including glucosinolate accumulation (Augustine & Bisht, 2015b; Mewis et al., 2005). At the core of our study is the hypothesis that XLGs could be the key signalling determinant in the plant's response to *S*. *sclerotiorum*, possibly by modulating the defence pathways and stress‐associated metabolites. Here, we report identification of multiple homologs of XLG‐encoding genes from the allotetraploid *B*. *juncea* and its progenitors, which are shaped by whole‐genome duplication and triplication events (Augustine et al., 2014). Using a series of molecular genetic and metabolite analyses, we demonstrated that specific silencing of *B*. *juncea XLG* homologs led to an altered susceptibility against *S*. *sclerotiorum* infection, which was further correlated with the altered expression of defence pathway genes and glucosinolate accumulation during pathogen infection. Our study is a novel endeavour to delineate the *XLG*‐mediated defence strategy in plants against *S*. *sclerotiorum*.

## 2 RESULTS

### 2.1 Identification and evolution of *B. juncea* XLG‐encoding genes

We first investigated the inventory and molecular evolution of *XLG* genes in the polyploid *Brassica* species. Using *AtXLG1–3* protein sequences as reference queries on the publicly available databases, multiple *XLG*‐like sequences were retrieved from the allotetraploid *B*. *juncea* (AB genome) and its progenitors, namely, *B*. *rapa* (A genome) and *B*. *nigra* (B genome). The diploid *B*. *rapa* and *B*. *nigra* genomes contain one *XLG1*, three *XLG2*, and two *XLG3* sequences each (Table 1). Subsequently, a total of two *XLG1*, five *XLG2*, and three *XLG3* sequences could be identified in *B*. *juncea*; the A and B subgenome‐specific homologs could be ascertained using the *B*. *rapa* and *B*. *nigra* gene sequences. The *XLG* genes identified in this study were named following the nomenclature adopted for *Brassica* genes (Østergaard & King, 2008).

**TABLE 1:** Summary of extra‐large G‐protein (XLG)‐encoding genes identified from *Brassica juncea* and its progenitor genomes

Name of gene	Gene ID in BRAD v2.0	Coding sequence (bp)	Protein (amino acids)	Amino acid identity with *Arabidopsis* orthologs (%)
***B. rapa* (A genome)**				
*BraXLG1‐A1*	Bra032166	2,577	859	85.9
*BraXLG2‐A1*	Bra011526 (Brara.A0038.1)^a^	2,448	816	76.8
*BraXLG2‐A2*	Bra017647	1,578	526	64.1
*BraXLG2‐A3*	Bra034623	2,505	835	73.7
*BraXLG3‐A1*	Bra023220	2,511	837	88.9
*BraXLG3‐A2*	Bra033865	2,514	838	88.3
***B. nigra* (B genome)**				
*BniXLG1‐B1*	BniB027874	2,604	868	84.7
*BniXLG2‐B1*	BniB032952	2,502	840	74.9
*BniXLG2‐B2*	BniB002451	2,541	847	73.6
*BniXLG2‐B3*	BniB048389	2,562	854	71.8
*BniXLG3‐B1*	BniB016337	2,523	841	88.5
*BniXLG3‐B2*	BniB042917	2,538	846	88.2
***B. juncea* (AB genome)** ^b^				
*BjuXLG1‐A1*	A04	2,577	859	85.8
*BjuXLG1‐B1*	B01	2,604	868	84.0
*BjuXLG2‐A1*	A01	2,478	826	75.9
*BjuXLG2‐A2*	A03	1,653	551	61.0
*BjuXLG2‐A3*	nd	nd	nd	nd
*BjuXLG2‐B1*	B05	2,547	849	75.0
*BjuXLG2‐B2*	B02	2,544	848	73.7
*BjuXLG2‐B3*	B03	2,577	859	72.2
*BjuXLG3‐A1*	A09	2,514	837	88.9
*BjuXLG3‐A2*	A05	2,508	836	87.7
*BjuXLG3‐B1*	B04	2,520	840	88.8
*BjuXLG3‐B2*	nd	nd	nd	nd

The sequences were obtained from BRAD database v2.0 (http://brassicadb.org/brad).

^a^ Full‐length sequence retrieved from Phytozome (https://phytozome.jgi.doe.gov/pz/portal.html).

^b^ Chromosomal location of *B. juncea XLG* genes obtained from BRAD database.

nd Full‐length sequences for *BjuXLG2‐A3* and *BjuXLG3‐B2* genes not determined from BRAD and Phytozome databases.

The full‐length coding sequence of *Brassica* *XLG*s ranged from 2,448 to 2,604 bp, encoding functional proteins of 816–868 amino acids (Table 1). Sequence alignment of the coding sequences revealed that the *Brassica XLG1*, *XLG2*, and *XLG3* homologs shared 91.1%–98.3%, 58.5%–98.4%, and 82.8%–99.2% identity within respective group members (Table S1). As expected, the *B*. *juncea XLG1*, *XLG2*, and *XLG3* genes shared a high sequence identity (>92.8%) with their corresponding *B*. *rapa* and *B*. *nigra* orthologs. Furthermore, analysis of the deduced amino acid sequences revealed that BjuXLG1, BjuXLG2, and BjuXLG3 proteins shared 84.0%–85.8%, 61.0%–75.9%, and 87.7%–88.9% identity with their corresponding AtXLG1, AtXLG2, and AtXLG3 proteins (Table 1). The BjuXLG proteins contain the predicted nuclear localization signal (NLS) and Cys‐rich domains at the N‐terminal region, while their C‐terminal region harbours the signature sequences of the five characteristic domains (G1–G5) required for guanine nucleotide binding (Figure S1; Ding et al., 2008).

**FIGURE 1:**
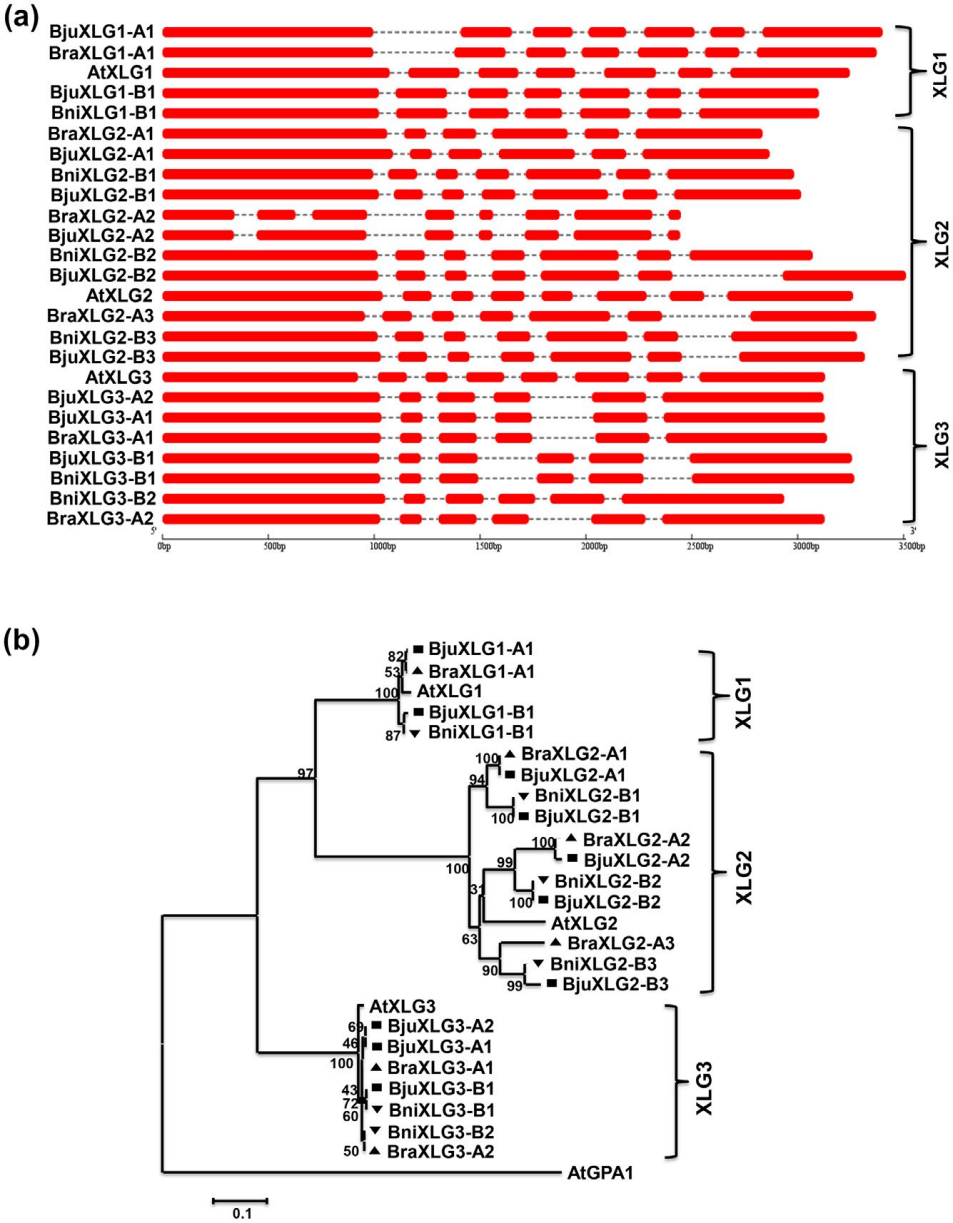
Gene organization and phylogenetic relationship of XLG‐encoding genes identified in *Brassica* species. (a) Gene structure of the identified *XLG* genes from *B*. *rapa* (Bra), *B*. *nigra* (Bni), and *B*. *juncea* (Bju), showing the arrangement of the exons (red boxes) and introns (dashed line), based on an alignment tool (http://gsds.cbi.pku.edu.cn/). (b) Phylogenetic analysis of the deduced XLG proteins from *B*. *juncea* (■ Bju) with XLG proteins from *B*. *rapa* (▲, Bra), *B*. *nigra* (▼, Bni), and *Arabidopsis* *thaliana* (At) was performed using the neighbour‐joining method in MEGA 6 (Tamura et al., 2013). The values shown above the branches represent the bootstrap percentage of replicate trees in which the associated proteins clustered together (500 replicates). The tree is drawn to scale, with branch length measured in the number of substitutions per site. The summary of the XLG‐encoding genes is provided in Table 1, following the nomenclature adopted for *Brassica* genes (Østergaard & King, 2008)

The presence of multiple *XLG‐*like sequences in the extant *Brassica* species led us to investigate the evolution of the *XLG* gene family by analysing their genomic structure and phylogenetic relationship. Analysis of genomic structure (http://gsds.cbi.pku.edu.cn/) showed that the *XLG* genes contain six to eight exons (Figure 1a). In comparison, the introns in *XLG* genes were highly divergent in their sizes and sequences, suggesting independent evolution and expansion of *XLG1*, *XLG2*, and *XLG3* genes in the *Brassica* lineage. To get a better insight into the expansion of the *XLG* gene family, a phylogenetic tree was constructed based on the multiple sequence alignment of full‐length protein sequences identified from *Brassica* species and their *A. thaliana* counterparts. Phylogenetic analysis showed that XLG proteins from the four plant genomes were clustered into distinct XLG1, XLG2, and XLG3 classes (Figure 1b). Within each class, the *B*. *juncea* XLG proteins were grouped with their closest *B*. *rapa* (A) and *B*. *nigra* (B) orthologs, supported by high bootstrap values. Notably, the higher branch lengths of XLG2 proteins on the phylogenetic tree suggest that the XLG2 proteins have diverged more compared to XLG1 and XLG3 proteins. Furthermore, analysis of synteny in the BRAD database (http://brassicadb.org/brad/) showed that *XLG2* homologs are retained in all three triplicated (least, moderately, and most fractionized) subgenomes, whereas homologs of *XLG1* and *XLG3* have experienced gene fractionation following the whole‐genome triplication event in the diploid *Brassica* species (Table S2). Overall, the whole‐genome triplication and gene fractionation (gene loss) events unique to the *Brassica* lineage have shaped both quantitative and structural divergence of the *XLG* genes.

### 2.2 Expression profiling of *B*. *juncea XLG* genes during plant developmental stages and in response to *S. sclerotiorum* infection

**FIGURE 2:**
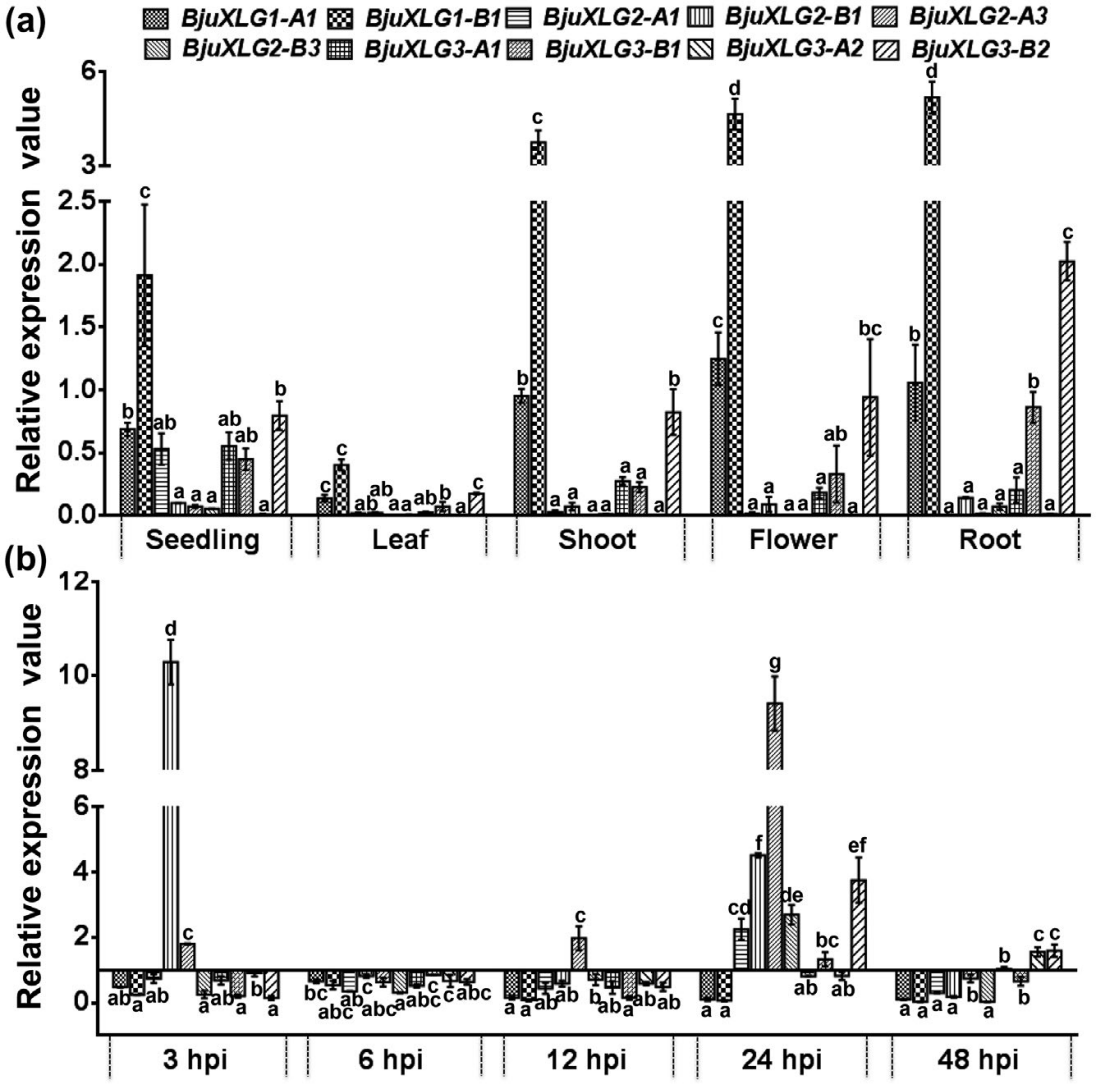
Expression analysis of *Brassica juncea XLG* homologs at different developmental stages and during *Sclerotinia sclerotiorum* infection. Expression patterns of *BjuXLG1*, *BjuXLG2*, and *BjuXLG3* homologs (a) in seedling (5 days), young leaf (1‐month‐old plant), shoot (1‐month‐old plant), unopened flower bud, and root (1‐month‐old plant), and (b) in response to *S*. *sclerotium* isolate Delhi‐1 (SSD1) infection from 3 to 48 hr postinfection (hpi). The relative expression value of *BjuXLG* genes was obtained by quantitative reverse transcription PCR analysis using gene‐specific primers (Table S5) and normalizing their expression against the endogenous controls *BjuActin* (set at 100) during plant developmental stages and *BjuTIP41* (set at 1) for SSD1 infection experiment (Chandna et al., 2012). Three independent experiments were performed, each with two technical replicates. Error bars represent ± *SE* of the mean. Different letters indicate significant differences among the homologs of *BjuXLG* genes for each developmental stage or time point separately, calculated using one‐way analysis of variance (*p* < 0.05) following Tukey's post hoc test

The three *XLG* genes of *Arabidopsis* display distinct spatiotemporal expression patterns during plant development (Figure S2). The multiplicity of *XLG* genes led us towards determining their expression patterns in *B*. *juncea*. Quantitative reverse transcription PCR (RT‐qPCR) analysis suggested that the *BjuXLG* genes were ubiquitously expressed and showed distinct expression patterns in tissue types representing different developmental stages. The expression patterns of the two *BjuXLG1* homologs were similar across the tested tissue types, although the abundance of *BjuXLG1‐B1* was higher than *BjuXLG1‐A1* (Figure 2a). The *BjuXLG3* homologs also showed distinct expression patterns, wherein *BjuXLG3‐B2* was the most abundant transcript across the tested tissue types. However, in comparison to the *BjuXLG1* and *BjuXLG3* genes, the *BjuXLG2* homologs had in general lower, yet distinct expression patterns during the plant developmental stages. Among the six *BjuXLG2* homologs, expression of only four genes could be detected, whereas the expression of *BjuXLG2‐A2* and *BjuXLG2‐B2* transcripts was found to be negligible in all the tested tissue types (Figure 2a). *BjuXLG2‐A1* showed higher expression in the seedling stage. Notably, for each of the *BjuXLG* genes, the B subgenome‐specific homologs showed a higher transcript accumulation compared to their A subgenome counterparts. This overlapping and distinct tissue‐specific expression pattern of multiple homologs suggests spatiotemporal regulation of *XLG* genes in *B*. *juncea*.

Furthermore, to determine whether *B*. *juncea* XLGs function in the defence response, cotyledons were inoculated with *S*. *sclerotiorum* isolate *Delhi1* (SSD1) and the expression pattern of the *BjuXLG* genes was analysed using RT‐qPCR at different time points (3–48 hr postinoculation [hpi]). The expression of *BjuXLG* genes was found to be altered differentially during SSD1 infection (Figure 2b). In general, both *BjuXLG1* homologs showed significant downregulation at different time points of infection. In contrast, *BjuXLG2* homologs exhibited increased transcript accumulation during SSD1 infection at one or more time points. For example, *BjuXLG2‐B1* and *BjuXLG2‐A3* were upregulated at as early as 3 hpi, *BjuXLG2‐A3* at 12 hpi, and all *BjuXLG2* homologs showed a strong up‐regulation at 24 hpi. Among the four *BjuXLG3* homologs, *BjuXLG3‐B2* showed strong upregulation at 24 hpi, whereas *BjuXLG3‐A1*, *BjuXLG3‐A2*, and *BjuXLG3‐B2* homologs were upregulated at the later stages of infection (48 hpi). Overall, these results suggest a possible involvement of *BjuXLG2* and *BjuXLG3* during *S. sclerotiorum* infection in *B*. *juncea*.

### 2.3 Development and molecular analysis of knockdown lines of *B. juncea XLG* genes

**FIGURE 3:**
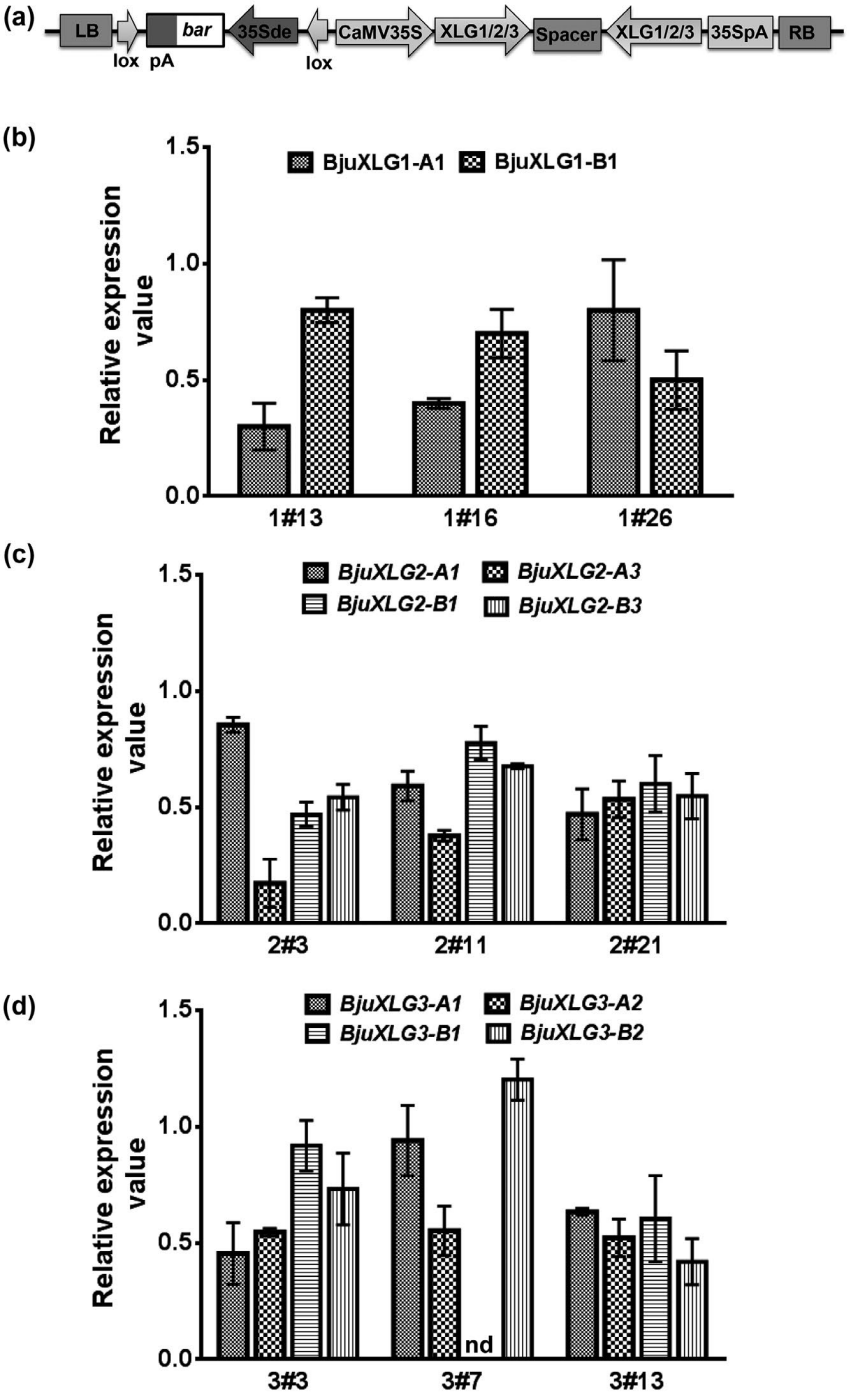
Molecular analysis of *Brassica juncea* XLG*‐*RNAi lines. (a) A representative T‐DNA map of BjuXLG‐RNAi construct. LB and RB, left and right T‐DNA borders, respectively; CaMV35S, 35S promoter of cauliflower mosaic virus; 35Sde, 35S promoter with double enhancers; Bar, fragment conferring resistance to the herbicide glufosinate; lox, lox site of the *cre*‐lox recombination system. The highly conserved 388, 309, and 371 bp fragments targeting the most conserved exon region of *BjuXLG1*, *BjuXLG2*, and *BjuXLG3* homologs, respectively, were cloned in sense and antisense orientations spanning the 409 bp region of the intron (used as spacer) of the native *BjuMYB28* (Augustine et al., 2013). The expression profile of (b) *BjuXLG1* homologs in BjuXLG1‐RNAi, (c) *BjuXLG2* homologs in BjuXLG2‐RNAi, and (d) *BjuXLG3* homologs in BjuXLG3‐RNAi lines. The expression was determined in 5‐day‐old seedlings in T_2_ generation plants of the representative single‐copy events. Vector control (VC) was used as the reference control (set at 1) and the values of each gene were normalized using *BjuActin* (Chandna et al., 2012). Three independent experiments were performed each with two technical repeats and error bars represent ± *SEM*

To study the roles of *BjuXLG* genes during pathogenesis of *S*. *sclerotiorum* isolate Delhi‐1 (SSD1), intron‐spliced hairpin RNAi (hpRNAi) constructs, independently targeting the *BjuXLG1*, *BjuXLG2*, and *BjuXLG3* genes, driven by the constitutive CaMV 35S promoter were developed (Figures 3a and S3; Table S3). A large number of *B*. *juncea* T_0_ transformants were generated using *Agrobacterium‐*mediated genetic transformation of hpRNAi constructs targeting *BjuXLG* genes along with the vector control (VC) construct (Table 2).

**TABLE 2:** Summary of BjuXLG‐RNAi lines developed in the current study. The binary vector pPZP200GW:lox‐bar (Augustine et al., 2013) was used to develop the BjuXLG‐RNAi constructs

Construct code	Target gene(s)	Target region (position from ATG)	No. of T_0_ lines developed	No. of single‐copy lines identified
BjuXLG1‐RNAi	*BjuXLG1‐A1*	1175–1563	28	11
	*BjuXLG1‐B1*			
BjuXLG2‐RNAi	*BjuXLG2‐A1*	1160–1469	21	11
	*BjuXLG2‐A2*			
	*BjuXLG2‐A3*			
	*BjuXLG2‐B1*			
	*BjuXLG2‐B2*			
	*BjuXLG2‐B3*			
BjuXLG3‐RNAi	*BjuXLG3‐A1*	1075–1446	30	14
	*BjuXLG3‐A2*			
	*BjuXLG3‐B1*			
	*BjuXLG3‐B2*			

To minimize any variation caused due to copy‐number and cosuppression of the inserted transgene, only T_0_ events showing single‐copy integration were selected. Basta (active ingredient glufosinate) segregation analysis in the germinated T_1_ progeny (3 resistant:1 susceptible) identified single‐copy events for each construct, which were propagated until the T_2_ generation (Table S4). The downregulation of *BjuXLG1*, *BjuXLG2*, and *BjuXLG3* homologs in single‐copy transgenic lines was analysed in 5‐day‐old T_2_ seedlings through RT‐qPCR using homolog‐specific primers (Table S5). Expression analysis showed variable levels of downregulation of different *BjuXLG* homologs in the selected single‐copy transgenic lines. Among the tested BjuXLG1‐RNAi lines, 1#13 and 1#16 showed approximately 20%–70% decrease of *BjuXLG1* homologs compared to the VC (set at 1) (Figure 3b). Similarly, the expression of most of the *BjuXLG2* homologs was downregulated from 20% to 80% in BjuXLG2‐RNAi lines 2#3, 2#11, and 2#21 as compared to the VC (Figure 3c). In BjuXLG3‐RNAi lines (3#3, 3#7, and 3#13), expression of *BjuXLG3* homologs was found to be downregulated 10%–60% compared to the VC (Figure 3d). Our results clearly show that there was a great deal of variation in silencing efficiency between transgenic lines and the target gene homologs in *B*. *juncea*.

### 2.4 Disease response of *B. juncea* XLG*‐*RNAi lines during infection of *S. sclerotiorum*

We further investigated the effect of *XLG* silencing in *B*. *juncea* on infection by *S*. *sclerotiorum*. Two independent single‐copy BjuXLG‐RNAi lines showing comparable transcript downregulation of the respective homologs of *BjuXLG1* (1#13, 1#16), *BjuXLG2* (2#11, 2#21), and *BjuXLG3* (3#7, 3#13) were selected. True leaves from T_2_ homozygous lines, along with VC plants, were used for inoculation with SSD1 mycelial plugs and the disease progression was compared from 24 to 48 hpi.

**FIGURE 4:**
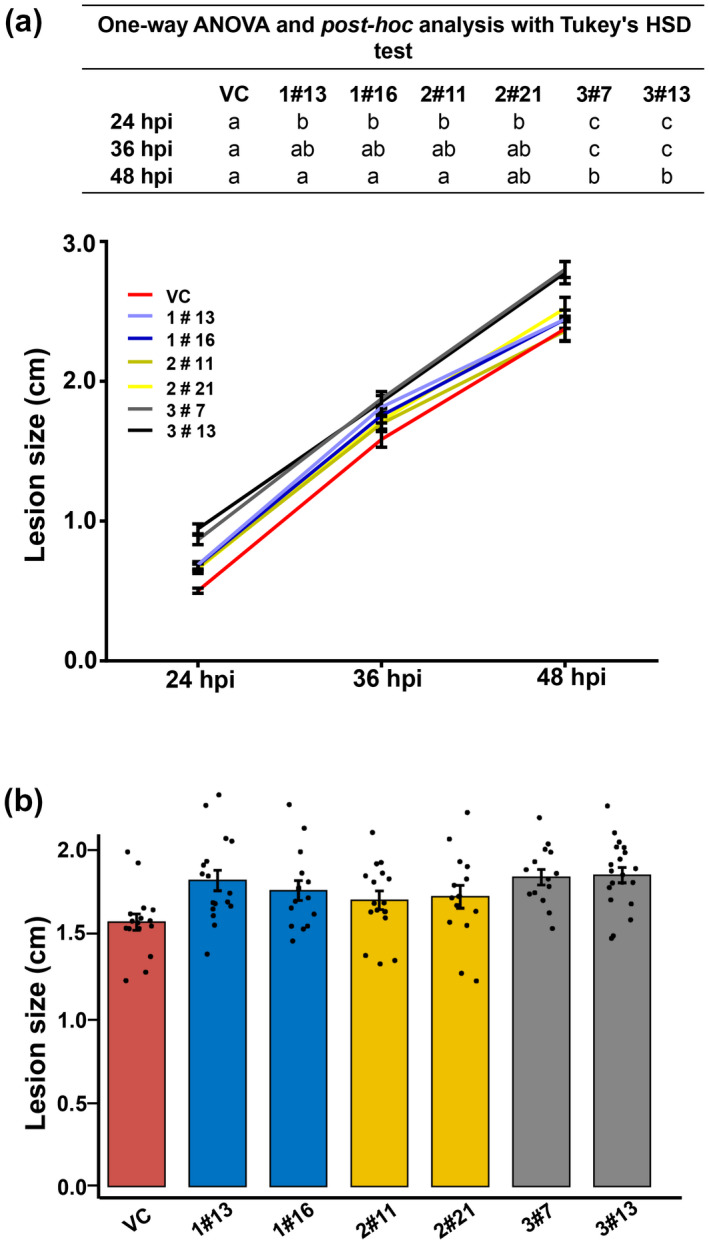
Disease response of *Brassica juncea* XLG‐RNAi lines inoculated with *Sclerotinia sclerotiorum*. (a) Lesion size of BjuXLG‐RNAi and vector control (VC) inoculated with *S*. *sclerotiorum* isolate Delhi 1 (SSD1) from 24 to 48 hr postinoculation (hpi). The experiment was repeated three times with similar results. Letters on top indicate values significantly different from each other as determined by one‐way analysis of variance following Tukey's post hoc test (*p* ≤ 0.05). (b) Jitter plot showing the mean lesion size in the leaves of BjuXLG‐RNAi and VC lines, inoculated with SSD1, at 36 hpi. Dots represent lesion size of independent progeny (replicates) of each RNAi line (*n* ≥ 10). The lines were colour coded as BjuXLG1‐RNAi (blue), BjuXLG2‐RNAi (yellow), BjuXLG3‐RNAi (grey), and VC (red)

Disease progression analysis showed a visible lesion size difference between transgenic lines and VC plants, which was apparent at 24 and 36 hpi and reached a maximum at 48 hpi (Figure 4a), suggesting that *B*. *juncea XLG* knockdown lines showed more rapid lesion expansion as compared to the VC plants. At 24 hpi, all the tested BjuXLG1‐RNAi, BjuXLG2‐RNAi, and BjuXLG3‐RNAi lines displayed significantly larger lesion size (*p* < 0.05) compared to the VC plants. However, at later time points (36 and 48 hpi), the lesion size in BjuXLG3‐RNAi lines was significantly higher than the BjuXLG1‐RNAi, BjuXLG2‐RNAi, and VC plants (Figure 4a,b). Thus, plants with suppression of *BjuXLG1* and *BjuXLG2* displayed enhanced disease susceptibility phenotype at the early stages of infection, whereas plants with *BjuXLG3* suppression showed a susceptible disease response throughout the SSD1 infection.

**FIGURE 5:**
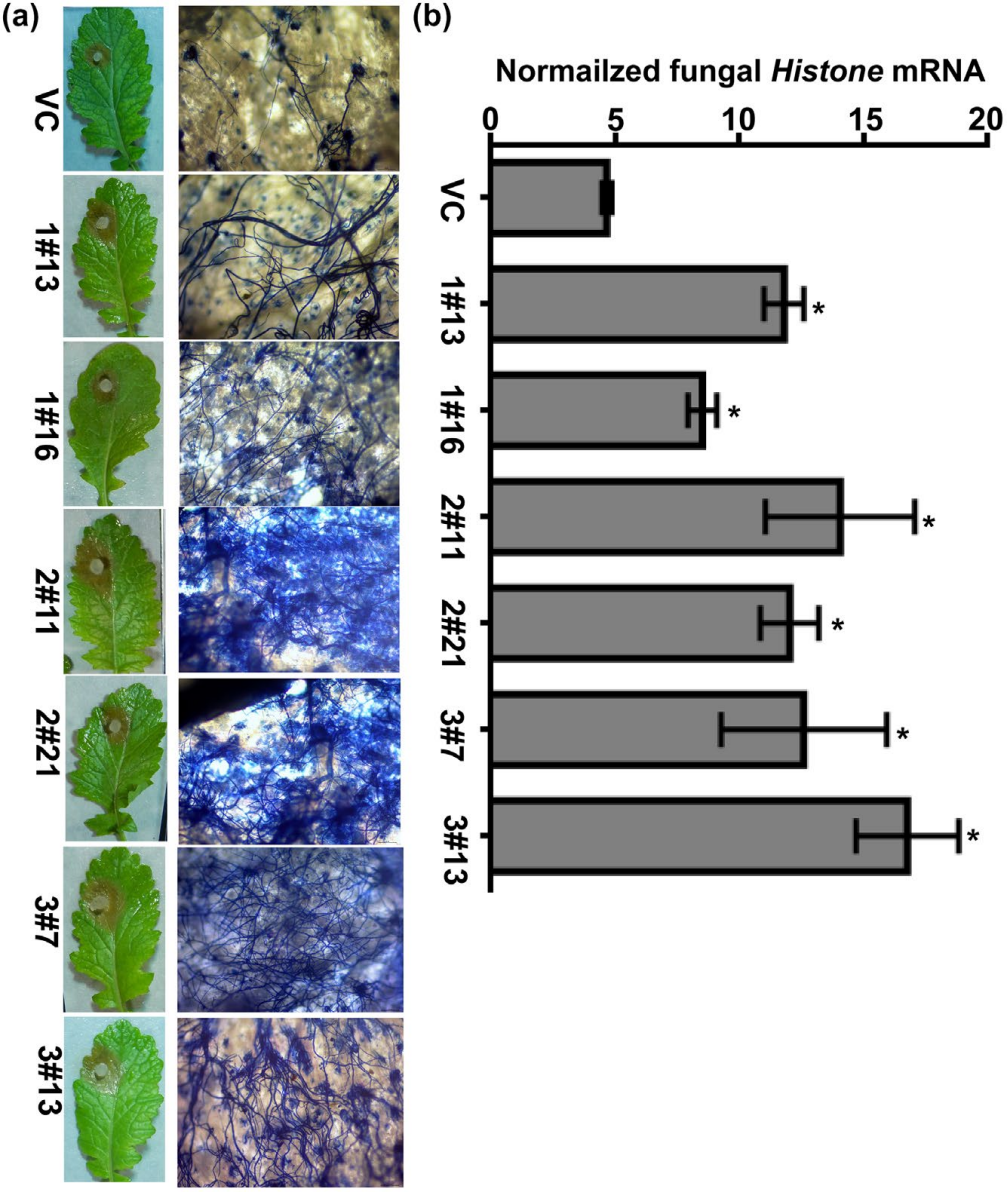
The assessment of *Sclerotinia sclerotiorum* (SSD1) fungal infection in *Brassica juncea* XLG‐RNAi lines. (a) The growth of *S*. *sclerotiorum* examined by trypan blue staining. Photographs were taken 24 hr postinoculation (hpi) and mycelial growth on stained leaves—was visualized with bright field microscopy. Bar indicates 20 µm. (b) Expression of *S*. *sclerotiorum* growth on BjuXLG‐RNAi and vector control (VC) plants after SSD1 infection (24 hpi) using quantitative reverse transcription PCR. The expression of *S*. *sclerotiorum* *Histone* mRNA was normalized against the constitutive *BjuTIP41* gene expression level. Three independent experiments were performed, each with two technical repeats. Error bars represent ± *SEM*. Asterisks (*) indicate significant differences (*p* < 0.05, Student's *t* test) between VC and BjuXLG‐RNAi lines

The hyphal growth of *S*. *sclerotiorum* in the infected leaf tissue was also examined by trypan blue staining at 24 hpi (Figure 5a). BjuXLG‐RNAi lines infected with SSD1 showed intense blue staining and the mycelial growth was densely arranged as compared to the VC plants. Additionally, quantitative fungal colonization on the inoculated leaves was also compared using the RT‐qPCR analysis. The expression of the *S*. *sclerotiorum* housekeeping gene *Histone* was significantly higher on BjuXLG1‐RNAi, BjuXLG2‐RNAi, and BjuXLG3‐RNAi lines compared to the VC plants (Figure 5b). These results indicate a positive role of *XLG* genes in mitigating *S*. *sclerotiorum* infection in *B*. *juncea*.

### 2.5 Expression of defence marker genes in *B. juncea* XLG*‐*RNAi lines during infection of *S. sclerotiorum*

**FIGURE 6:**
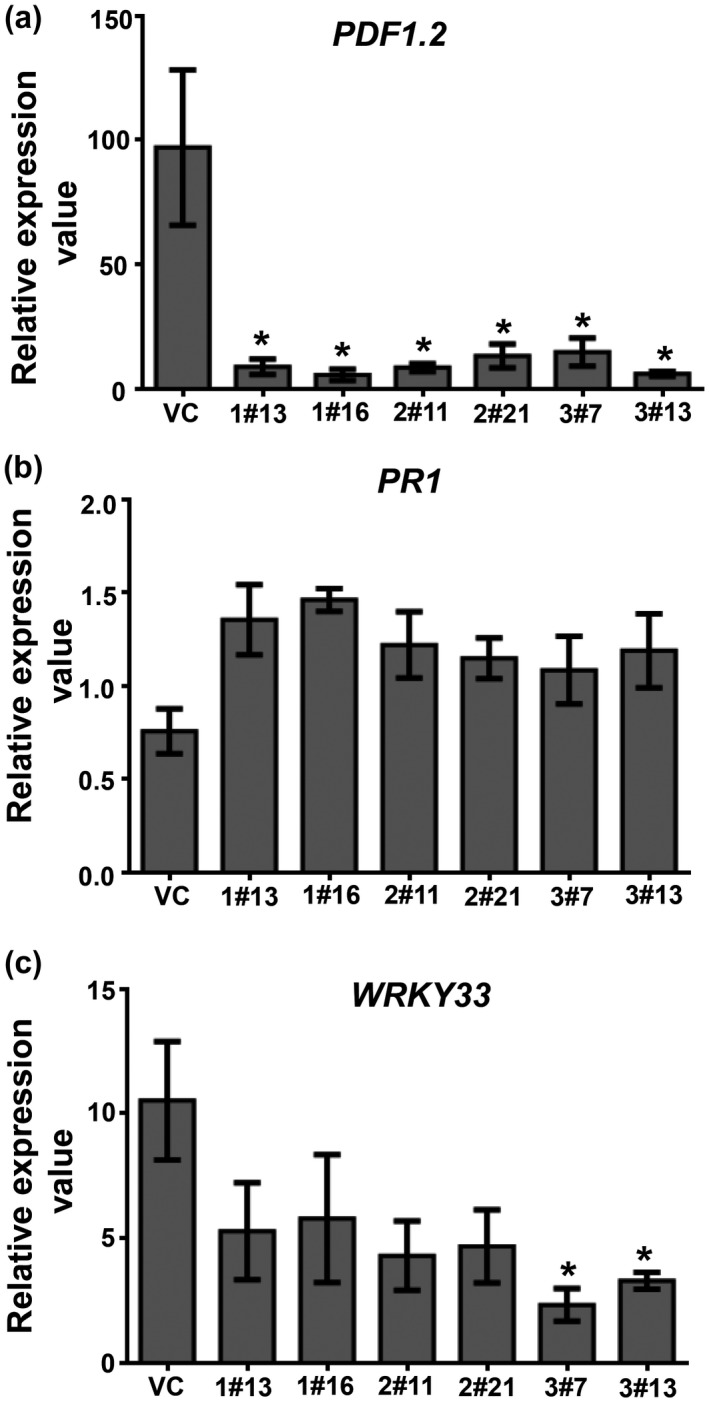
Effects of *Sclerotinia sclerotiorum* fungal infection on expression of defence marker genes in *Brassica juncea* XLG‐RNAi lines. Fold expression value of defence marker genes (a) *PDF1.2*, (b) *PR1*, and (c) *WRKY33* in *B*. *juncea* XLG‐RNAi lines and vector control (VC) plants in *S. sclerotiorum*‐inoculated (24 hr postinoculation) leaf sample with respect to the corresponding nontreated mock samples. Transcripts levels were normalized against the constitutive *BjuTIP41* gene expression level (VC mock set at 1). Three independent experiments were performed, each with two technical repeats. Error bars represent ± *SEM*. Asterisks (*) indicate significant differences (*p* < 0.05, Student's *t* test) between VC and BjuXLG‐RNAi lines

To investigate whether the suppression of *B*. *juncea XLG* genes leads to expression changes in defence‐marker genes, we examined the transcription abundance of the commonly used *PDF1.2* (JA pathway) and *PR‐1* (SA pathway) genes using RT‐qPCR (Table S5). During the noninfected (mock) condition, the abundance of the selected defence‐marker genes in the XLG‐RNAi lines was found to be comparable to the vector control plants (Figure S4). The expression of these defence‐marker genes was analysed in SSD1‐infected leaf samples at 24 hpi, and the fold change was compared with mock plants (set at 1). The upregulation of *PDF1.2* was significantly compromised in BjuXLG‐RNAi lines compared to the VC plants, showing c.90‐fold transcript upregulation (Figure 6a). In contrast, expression of *PR‐1* was not altered in the BjuXLG‐RNAi lines and VC plants during SSD1‐infected conditions (Figure 6b), suggesting that *S*. *sclerotiorum* infection follows an SA‐independent route in plants.

The mRNA accumulation of another defence marker gene, *WRKY33*, regulating phytoalexin biosynthesis in *Arabidopsis* (Mao et al., 2011), was also evaluated. A few earlier reports have documented the key role of *WRKY33* in resistance against *S*. *sclerotiorum* in oilseed rape, *B*. *napus*, possibly by activation of JA‐ and SA‐mediated defence response (Liu et al, 2018; Wang et al., 2014). In comparison to the VC plants, the BjuXLG‐RNAi lines showed comparably lower upregulation of *WRKY33* during SSD1 infection, wherein a significant inhibition of *WRKY33* transcription was observed for the BjuXLG3‐RNAi lines (Figure 6c). The altered expression of the key defence marker genes (*WRKY33* and *PDF1.2*) in *BjuXLG‐RNAi* lines during SSD1 infection indicates a direct role of *B*. *juncea* *XLG* genes in the *S*. *sclerotiorum* defence response.

### 2.6 Glucosinolate accumulation in *B. juncea* XLG‐RNAi lines during *S. sclerotiorum* infection

**FIGURE 7:**
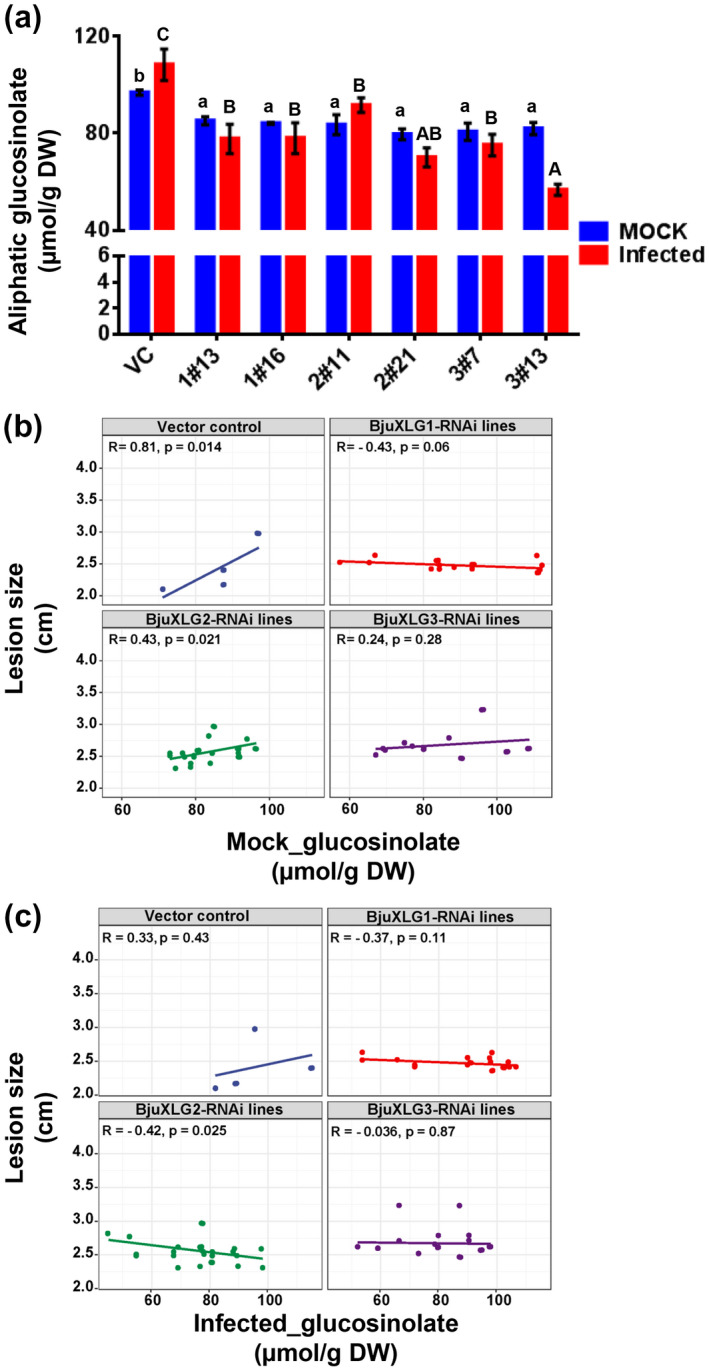
Correlation of aliphatic leaf glucosinolates with disease severity of *Brassica juncea* XLG‐RNAi lines inoculated with *Sclerotinia sclerotiorum*. (a) Aliphatic glucosinolate content (µmol/g dry weight) was determined in 20‐day‐old BjuXLG‐RNAi lines inoculated with SSD1 (3 hr postinoculation). Data represent the mean ± *SE* (*n* = 4) for each genotype. Letters above bars indicate values significantly different from each other as determined by one‐way analysis of variance followed by Tukey's post hoc test (*p* ≤ 0.05). Different letters indicate significant differences among plant genotypes, lower case letters for mock (untreated) plants and upper case letters for SSD1 infected plants. (b,c) Linear regression analysis between lesion size (cm) of *B*. *juncea* XLG‐RNAi lines with leaf aliphatic glucosinolates (μmol/g dry weight) present in (b) mock and (c) SSD1‐infected conditions. Statistical analysis was performed using RStudio v. 3.6.3

Because *Brassica* species possess high amounts of specialized defence metabolites, glucosinolates, we were curious to investigate if suppression of *XLG* genes in *B*. *juncea* had any effects on glucosinolate levels. Glucosinolate content and composition was estimated at an initial time point of SSD1 infection in intact leaves of BjuXLG1‐RNAi, BjuXLG2‐RNAi, and BjuXLG3‐RNAi lines (Table S6). The most abundant glucosinolates in *B*. *juncea* leaves were those belonging to the aliphatic glucosinolates (allyl/sinigrin, 3‐butenyl, and 4‐pentenyl), whereas indolic (4OH‐I3M [4‐hydroxyindol‐3‐ylmethyl], I3M [indol‐3‐ylmethyl], and 4MO‐I3M [4‐methoxyindol‐3‐ylmethyl]) glucosinolates were present in minor fractions. The concentration of aliphatic glucosinolates in the VC line was found to be 96.77 ± 0.06 µmol/g dry weight (DW) in mock condition, which increased to 108.29 ± 6.43 µmol/g DW at an early time point (3 hpi) of pathogen infection (Table S6 and Figure 7a). We observed that the accumulation of aliphatic glucosinolates in BjuXLG‐RNAi lines was significantly lower than in the VC plants in both infected and mock plants. It was noteworthy that the accumulation of aliphatic glucosinolates in the BjuXLG1‐RNAi, BjuXLG2‐RNAi (except 2#11), and BjuXLG3‐RNAi lines showed marked reduction of leaf glucosinolates after SSD1 infection, with silencing of *BjuXLG3* providing a more drastic effect. The altered defence response of the BjuXLG‐RNAi lines thus seems to have resulted from the altered accumulation of aliphatic glucosinolates (Figure 7a). However, the levels of indolic glucosinolates in mock and SSD1‐inoculated plants did not change among BjuXLG‐RNAi lines and VC plants (Table S6).

To investigate the effect of plant type (vector control and BjuXLG‐RNAi lines) and treatments (mock and SSD1 infection) on the alteration of aliphatic, indolic, and total glucosinolates, two‐way analysis of variance (ANOVA) was performed. The analysis indicated that plant type had significant impact on the levels of all glucosinolates, whereas the effect of treatment was significant for aliphatic and total glucosinolates (Table 3). The interaction between plant type and treatment further suggested that aliphatic glucosinolates had a predominant effect during the *B*. *juncea*–SSD1 interaction. We examined the correlation between the disease severity (lesion size) of *B*. *juncea* XLG‐RNAi lines and aliphatic glucosinolate content during both noninfected and postinfection conditions. Using the simple linear model, lesion size showed a positive correlation with leaf aliphatic glucosinolate content of the noninfected VC as well as BjuXLG‐RNAi (except BjuXLG1‐RNAi) lines (Figure 7b). However, the lesion size of BjuXLG‐RNAi showed a negative correlation with leaf aliphatic glucosinolate after SSD1 infection, which was in contrast to the VC plants (Figure 7c). Furthermore, BjuXLG2‐RNAi lines had a more profound effect on lesion size in response to *S*. *sclerotiorum* infection. Overall, our data indicated that the knockdown of *B*. *juncea XLG* genes leads to differential alteration of leaf glucosinolate accumulation vis‐à‐vis altered susceptibility against the necrotrophic pathogen *S*. *sclerotiorum*.

**TABLE 3:** Summary of two‐way analysis of variance of the impact of plant type (vector control, BjuXLG1‐RNAi lines, BjuXLG2‐RNAi lines, and BjuXLG3‐RNAi lines) and treatment (noninoculated mock and *Sclerotinia sclerotiorum*‐inoculated) on the levels of aliphatic, indolic, and total glucosinolates

Factors	*df*	Aliphatic glucosinolate		Indolic glucosinolate		Total glucosinolate	
		*F*	*p*	*F*	*p*	*F*	*p*
Plant type	6	23.533	**<0.001** ***	2.748	0.021*	23.078	**<0.001** ***
Treatment	1	9.512	0.003**	0.169	0.683	9.171	0.004**
Plant × Treatment	6	7.227	**<0.001** ***	3.164	0.01*	6.934	**<0.001** ***

Note

Significant effects are presented in bold.

* *p* < 0.05

** *p* < 0.01

*** *p* < 0.001.

## 3 DISCUSSION

*Brassica* crops suffer a huge yield loss due to various biotic insults, including pests and pathogens. Among them, sclerotinia stem rot incited by *S*. *sclerotiorum* causes a huge yield loss to *B*. *juncea*, with no known source of durable resistance identified to date. In this study, we investigated the role of G‐protein signalling during *Brassica–Sclerotinia* interaction and highlighted a key role of plant lineage‐specific XLG proteins in plant immunity against the necrotrophic pathogen.

### 3.1 XLG‐encoding genes in the allotetraploid *B. juncea* are shaped by differential gene retention and distinct expression patterns

Early findings in *Arabidopsis* and rice favoured the opinion that the core components of heterotrimeric G‐proteins signalling in plants are far less diverse than present in animals (Temple & Jones, 2007). However, the discovery of the noncanonical XLGs and type III Gγ proteins have expanded the diversity and plasticity of plant G‐protein signalling to regulate multiple biological processes and environmental signals (Ding et al., 2008; Lee & Assmann, 1999; Trusov & Botella, 2016). This opinion was further changed when multiple members of G‐protein subunit genes were reported in the palaeopolyploid genome of soybean (Bisht et al., 2011; Choudhary et al., 2011). Because >50% of plant species are known to be polyploids, the multiplicity of plant G‐protein components is now a norm.

Elucidating the molecular basis of G‐protein‐controlled phenotypes in *Brassica* crop species, compared to *Arabidopsis*, has been complicated by their inherent polyploidy and complex genomic architecture. Comparative mapping and sequence‐level studies confirmed the existence a *Brassica* lineage‐specific whole‐genome triplication event after their split from *Arabidopsis* about 13–17 million years ago (Franzke et al., 2011; Lysak et al., 2005), followed by large‐scale chromosomal rearrangements (genome‐fractionation) during diploidization and a recent interspecific hybridization (Mun et al., 2009; Wang et al., 2011). Thus, the polyploid *Brassica* species contain multiple homologs of both canonical and noncanonical G‐protein genes, suggesting the presence of a complex G‐protein signalling network.

The allotetraploid *B. juncea* genome has retained two to six homologs of the three *XLG* genes (Figure 1 and Table 1), resulting from the differential gene loss phenomenon in the triplicated subgenomes. Our molecular analysis further indicated that the *BjuXLG* genes are evolutionary conserved and probably evolved through hybridization of two relatively simple *B*. *rapa* and *B*. *nigra* genomes, while retaining sequence conservation following allopolyploidization. The presence of a distinct N‐terminal region and Gα‐like C‐terminal region confirmed structural conservation of XLG proteins in the core Brassicaceae. However, variation in exon‐intron organization and phylogenetic analysis suggests that *Brassica* lineage‐specific XLG2 proteins have diverged significantly compared to the XLG1 and XLG3 proteins during evolution (Figure 1). The differential gene retention and sequence divergence of the homologs of three *XLG* genes could have a significant consequence on their gene expression patterns and subfunctions in the polyploid *B*. *juncea*.

Accumulated data on gene expression in allopolyploids paints a picture of a highly perturbed transcriptomic network including expression level dominance, silencing of duplicated genes, bias in homeolog expression, and sub‐genome dominance (Renny‐Byfield & Wendel, 2014). These expression alterations accompanying polyploidy may also be important to the phenotypes and be responsive to environmental cues. The distinct spatiotemporal and condition‐specific expression patterns of multiple *BjuXLG* genes were evident in *B*. *juncea*, wherein *BjuXLG1* homologs have retained expression dominance during plant growth and development stages, whereas *BjuXLG2* and *BjuXLG3* homologs seem to be important during plant–pathogen interaction (Figure 2). Expression dominance of B subgenome homologs was also obvious in *B*. *juncea* tissue types, whereas there was little evidence of expression bias from either of the subgenomes during SSD1 infection. Expression dominance among different members of the core G‐protein complex has also been reported earlier in the polyploid soybean and *Brassica* species (Arya et al., 2014; Bisht et al., 2011; Choudhury et al., 2011; Kumar, Sharma, et al., 2017). The distinct expression patterns of *BjuXLG*s suggest that both tissue‐ and condition‐specific functional combination(s) of XLG proteins and Gβγ dimer may exist in the allotetraploid *B*. *juncea* to control diverse growth and development phenotypes. Similarly, in the model plant *Arabidopsis*, in which canonical Gα and Gβ proteins are present, the functional selectivity of G‐protein heterotrimer is reported to be under the tight control of divergent XLG and Gγ proteins (Trusov & Botella, 2016; Trusov et al., 2006; Urano et al., 2016).

### 3.2 Suppression of *BjuXLG* genes results in enhanced susceptibility to *S. sclerotiorum*

In *Arabidopsis* among the three *XLG* genes, *XLG2* is known to play a key role in resistance against the hemibiotrophic bacterium *P*. *syringae* and *XLG3* plays a part in resistance against the necrotrophic pathogen *Fusarium* *oxysporum*, whereas *XLG1* does not seem to have any role in the defence response (Maruta et al., 2015; Zhu et al., 2009). Differential transcript regulation of the diverse *XLG* genes in *B*. *juncea* offered insights into the involvement of specific *BjuXLG* genes during *S*. *sclerotiorum* infection. Significant downregulation of most of the *BjuXLG1*, *BjuXLG2*, and *BjuXLG3* homologs in RNAi lines was quite notable in this study. Generation of functional gene knockouts is quite a challenging task in polyploids owing to the selection of target region, copy number, and location of T‐DNA integration, which often leads to variation in gene silencing efficiency across transgenic lines. To the best of our knowledge and years of expertise with polyploid crops, the RNAi silencing strategy seems to be the most favourable strategy to knock down multiple target genes simultaneously in a polyploid crop species (Augustine & Bisht, 2019).

A clear correlation was observed between the transcript levels and disease phenotypes in leaves expressing altered levels of *BjuXLG* genes (Figures 3 and 4). The data are suggestive of the fact that, at an early stage of *S*. *sclerotiorum* infection, suppression of all three *BjuXLG* genes leads to profuse disease development in *B*. *juncea*, which was also evident from higher accumulation of fungal mass in the BjuXLG‐RNAi lines (Figure 5). Higher susceptibility and more necrosis confirmed the key role of *XLG* genes during the *Brassica–Sclerotinia* interaction. However, at a later stage of infection, the role of *BjuXLG3* homologs seems more evident (Figure 4). These data are supported by their expression analysis, where *BjuXLG2* and *BjuXLG3* homologs were upregulated at 24 hpi while only *BjuXLG3* homologs showed a significant upregulation at the later stage (48 hpi) of SSD1 infection in *B*. *juncea* (Figure 2). This temporal‐specific expression pattern of *XLG*s and disease response can be attributed to the nature of *S*. *sclerotiorum,* which has a transient hemibiotrophic stage during the early infection phase before the onset of the predominant necrotrophic phase (Williams et al., 2011), and this feature may trigger highly dynamic host defence responses at different stages of pathogen infection. Overall, our data suggest that *BjuXLG*s have redundant functions in *Brassica–Sclerotinia* interaction, and *BjuXLG3* plays a predominant role in resistance against *S*. *sclerotiorum* infection in *B*. *juncea*.

### 3.3 *BjuXLG* suppression leads to altered expression of defence marker genes

Previous studies in *A*. *thaliana* have shown that *XLG*s are positive regulators of resistance to biotrophic pathogens that largely trigger SA‐responsive genes, but no such information is available about necrotrophic pathogens (Maruta et al., 2015; Zhu et al., 2009). The role of JA/ET phytohormones in providing defence against *S*. *sclerotiorum* is also documented (Chen et al., 2021; Guo & Stotz, 2007). In *B. napus*, constitutive overexpression of *WRKY33* was found to provide resistance against *S*. *sclerotiorum* by modulating the expression of the JA/ET marker *PDF1.2* (Wang et al., 2014). To strengthen our understanding of XLG‐mediated defence responses in *B*. *juncea*, the screening of defence marker genes was analysed. The highly compromised expression of *PDF1.2* and *WRKY33* in BjuXLG‐RNAi lines during *S*. *sclerotiorum* infection (Figure 6) in *B*. *juncea* suggests that the *BjuXLGs* are important G‐protein signalling nodes upstream of these defence marker genes. Although the SA‐mediated defence response against *S*. *sclerotiorum* has been documented earlier (Guo & Stotz, 2007), the expression pattern of the SA pathway gene *PR‐1* was not altered during pathogen infection in our study, thereby indicating that *B*. *juncea* predominantly uses the JA‐dependent defence response against *S*. *sclerotiorum*.

WRKY33 is a pathogen‐inducible transcription factor whose expression is regulated by the pathogen‐responsive mitogen‐activated protein kinase (MPK3/MPK6) cascade in *A*. *thaliana* (Mao et al., 2011). It is known that in *Arabidopsis* WRKY33 impacts the biosynthesis of the indolic phytoalexin camalexin, the major phytoalexin, which probably contributes to resistance towards *S*. *sclerotiorum* (Stotz et al., 2011). Thus, the observed reduction of *WRKY33* expression in BjuXLG‐RNAi lines might lead to compromised accumulation of *Brassica*‐specific phytoalexins, which in turn lead to the observed enhanced susceptibility. Alternatively, WRKY33 can also affect other resistance mechanisms that are not dependent on specialized metabolites.

### 3.4 Susceptibility to *S. sclerotiorum* in XLG‐RNAi lines is correlated with aliphatic glucosinolate levels

The species belonging to Brassicaceae family contain glucosinolates that, along with their hydrolysis products, play important roles in plant protection against pests and pathogens (Chen et al., 2020; Hopkins et al., 2009; Kumar, Augustine, et al., 2017; Sotelo et al., 2015). Several studies suggest that both glucosinolate content and composition are altered on fungal infection (Abuyusuf et al., 2018; Robin et al., 2017). The necrotrophic fungus *S*. *sclerotiorum* causes more severe tissue damage than biotrophs, thereby making it more exposed to glucosinolates and their hydrolysis products (Kliebenstein, 2004). It has been noted that *Arabidopsis* mutants deficient in aliphatic or indolic glucosinolate biosynthesis are hypersusceptible to *S*. *sclerotiorum* (Chen et al., 2020; Stotz et al., 2011).

Glucosinolate levels are known to be positively correlated with oilseed rape (*B*. *napus*) resistance to *S*. *sclerotiorum* (Abuyusuf et al., 2018; Sotelo et al., 2015). An earlier study in *B*. *juncea* also highlighted that biotic elicitors and mechanical damage modulate glucosinolate accumulation (Augustine & Bisht, 2015b). In the current study, we observed that tissue damage by *S*. *sclerotiorum* infection induced glucosinolates in *B*. *juncea*, at least during the early time point of infection (Table S6). However, glucosinolate status was compromised in *XLG* knockdown plants (Table S6), demonstrating a possible cross‐talk between G‐protein and glucosinolate pathways. The accumulation of the predominant aliphatic (allyl and 3‐butenyl) glucosinolates was significantly compromised in the leaves of BjuXLG‐RNAi lines after SSD1 infection (Figure 7a; Table S6), whereas the indolic glucosinolate pool was marginally altered on pathogen infection. The correlation analysis provided an idea about the interplay between *S*. *sclerotiorum*‐induced disease severity and aliphatic glucosinolates, as lesion size was negatively correlated with leaf aliphatic glucosinolate content, an indication of XLG‐dependent resistance to *S*. *sclerotiorum* in *B*. *juncea* (Figure 7b,c). A previous study in *B*. *juncea* also demonstrated that the growth of *S*. *sclerotiorum* was negatively correlated with aliphatic glucosinolates, specifically the methyl‐sulphinyl alkyl glucosinolates (e.g., glucoraphanin and glucoalyssin), but not with the indolic glucosinolates (Augustine & Bisht, 2015a). The sugar‐insensitive RGS1 (Regulator of G‐protein Signalling 1) mutant of the *Arabidopsis* *rgs1* is also known to accumulate lower aliphatic glucosinolates, suggesting glucose signalling positively regulates glucosinolate biosynthesis via the G‐protein pathway (Miao et al., 2013).

Overall, our findings established that the plant lineage‐specific XLGs are potential signalling regulators in the *Brassica–Sclerotinia* interaction, a phenomenon not explored or described earlier. We also strengthened our understanding of the XLG‐dependent plant immune response in the core Brassicaceae family through the involvement of glucosinolates. Further experiments with altered levels of additional components of G‐protein signalling and identification of downstream components would help to shed light on the exact mechanisms of conserved and lineage‐specific G‐protein signalling involved during plant–pathogen interactions.

## 4 EXPERIMENTAL PROCEDURES

### 4.1 Plant material and growth conditions

*B. juncea* 'Varuna' was grown in an environmentally controlled growth chamber (Conviron, Canada) at 24 ± 1 °C light and 18 ± 1 °C dark with 10 hr light/14 hr dark regimes and light intensity of 260–300 µmol⋅m^−2^⋅s^−1^. Plants were grown in sand: soilrite mixture (1:2) and watered with nutrient medium twice a week. *B. juncea* transgenic lines were grown under the contained net‐house during the rabi growing season (October–April), as per the guidelines laid out by the Department of Biotechnology, Government of India.

### 4.2 Identification and phylogenetic analysis of *XLG* genes from *Brassica* species

The XLG‐encoding genes from *B*. *juncea*, *B*. *rapa*, and *B*. *nigra* were identified by performing BLAST analysis using AtXLG proteins as queries in the BRAD v. 2.0 (http://brassicadb.org/brad) and Phytozome v. 10.1 (https://phytozome.jgi. doe.gov/pz/portal.html) databases using stringent cut‐off values (E‐value = 0). To study evolutionary relationships, the identified XLG protein sequences were aligned by ClustalW and the phylogenetic tree constructed using the neighbour‐joining method, adopting the complete depletion option of the gaps with 500 replicated bootstrap value using MEGA 6.0 (Tamura et al., 2013). Genomic structures showing exon and intron boundaries of *XLG* genes were identified by gene structure display server (http://gsds.cbi.pku.edu.cn/).

### 4.3 Gene expression analysis

Different developmental tissues of *B*. *juncea* including seedling (5 days old), young leaf (1‐month‐old plant), shoot (1 month), flower bud (3–5 mm), and root (1‐month‐old plant) were collected and frozen immediately in liquid nitrogen. Five‐day‐old T_2_ seedlings of XLG‐RNAi lines were used for determining the expression of *BjuXLG* homologs.

Total RNA was isolated using a Spectrum Plant Total RNA Kit (Sigma Aldrich). cDNA was synthesized from approx. 2 µg of RNA using a High Capacity cDNA Reverse Transcription Kit (Applied Biosystems). RT‐qPCR analysis of the 1:20 diluted cDNA was performed using gene‐specific primers and iTaq universal SYBR Green super mix (Bio‐Rad), according to the manufacturer's instructions, in a CFX96 Real Time System (Bio‐Rad). *Actin* and *TIPS* genes of *Brassica* origin were used as endogenous controls following Chandna et al. (2012) (Table S5). Three independent biological samples, with two technical replicates each, were analysed to derive the conclusions.

### 4.4 Generation of *XLG* knockdown transformation constructs

For developing RNAi constructs, 388, 309, and 371 bp fragments targeting the most conserved exon region of *BjuXLG1*, *BjuXLG2*, and *BjuXLG3* homologs, respectively, were amplified using gene‐specific primers from cDNA and cloned into the pENTR/D‐TOPO vector (Table S5, Figure S3). These fragments were sequenced and mobilized into the pPZP200GW:lox‐bar binary vector (Augustine et al., 2013) in both sense and antisense orientations under the control of a constitutive CaMV 35S promoter, using Gateway‐based cloning. All constructs were transformed into *Agrobacterium tumefaciens* GV3101 using the freeze‐thaw method (Nishiguchi et al., 1987) and subsequently transformed into *B*. *juncea* 'Varuna' as per the protocol of Augustine et al. (2013). Briefly, approx. 1 cm explants from hypocotyls of 5‐ to 6‐day‐old seedlings were incubated in preculture medium supplemented with 1 mg/L each of NAA and BAP (N1B1 medium) for 18–24 hr, with constant shaking at 100 rpm. Subsequently, the explants were infected (30 min) and cocultivated overnight with transformed *Agrobacterium* culture in N1B1 medium at 23 ± 1 °C at 100 rpm. The explants after washing with Augmentin (200 mg/L) were plated on the shoot‐induction medium supplemented with AgNO_3_ (3.4 mg/L) and regenerated shoots were subsequently transferred to the rooting medium containing IBA (2 mg/L) with Basta (glufosinate at 10 mg/L) as the selection agent. Regenerated shoots were transferred to the rooting medium containing the selection agent, and the well‐rooted plants were transferred directly into the soil during the growing season in a containment net‐house facility according to the guidelines of the Department of Biotechnology, Government of India.

### 4.5 *Brassica* plant inoculation with *S. sclerotiorum*

Five‐day‐old mycelia of 4 °C preserved SSD1 fungal strain were cultured on fresh potato dextrose agar and grown for 3 days. Agar plugs (5 mm diameter) were excised from edges of growing colonies and upended onto adaxial surface of the three‐ to four‐leaf stage *B*. *juncea* plant. High humidity (>90%) and dark conditions were maintained during the infection period. The lesion size was calculated at 24, 36, and 48 hpi after the appearance of a clear measurable lesion using the formula:

$$\text{Lesion}\;\text{size}=\left(a+b\right)/2$$

where *a* and *b* represent the long and short diameters of the lesion, respectively. Every experiment was randomized and repeated at least three times with multiple technical replicates. The SSD1‐infected samples were harvested from 3 to 48 hpi for expression analysis of *BjuXLG* homologs and defence marker genes.

### 4.6 Histochemical staining and assessment fungal infection level

The growth of *S*. *sclerotiorum* in leaves of transgenic and wildtype plants was examined by trypan blue staining. The leaves were first cleared in acetic acid:ethanol solution (1:3) for 12 hr, followed by acetic acid:ethanol:glycerol (1:3:1) for 2 hr, and then stained in 0.01% trypan blue solution. Quantification of *S*. *sclerotiorum* mRNA in inoculated leaves was according to Chen et al. (2020). Total RNA was isolated from the infected leaf using the Spectrum Plant Total RNA Kit (Sigma Aldrich). For relative quantification of fungal colonization, the amount of normalized fungal mRNA was calculated by the ratio of fungal *Histone* gene expression to *B. juncea TIP41* gene expression (Table S5).

### 4.7 Glucosinolate estimation using HPLC

Leaf glucosinolates in BjuXLG*‐*RNAi lines under mock and 3 hpi of SSD1 were determined as desulpho‐glucosinolates following an established protocol (Augustine et al, 2013). Briefly, glucosinolates were extracted from 10 mg of lyophilized leaf in 1 ml of 70% methanol with sinalbin added as an internal standard (sinalbin was a kind gift from Dr Michael Rachielt, Max Plank Institute for Chemical Ecology, Jena, Germany). Desulfation of glucosinolates was performed overnight using purified sulfatase (25 mg/ml; Sigma‐Aldrich) on a DEAE Sephadex‐A25 column, and desulfo‐glucosinolates were eluted with 1 ml of HPLC‐grade water and 10 µl of eluent was analysed in a Shimadzu Nexera X2 UHPLC device (Shimadzu Corporation). The programme was set at a solvent B (acetonitrile) gradient of 1%–19%, with respect to solvent A (water) through a 30 min cycle with flow rate of 1 ml/min using a Luna C18 reverse‐phase column (150 × 4.6 mm, 0.5 mm i.d.) and glucosinolate compounds were detected at 229 nm. Concentration of glucosinolate fractions, expressed as µmoles per gram dry weight of tissue (µmol/g DW), was calculated relative to the internal standard peak and applying their relative response factors reported earlier (Brown et al., 2003). Data given are the mean of four independent measurements ± *SE*.

### 4.8 Statistical analyses

Means of groups were compared by ANOVA Tukey's multiple comparison test, performed using SPSS statistic v. 23.0 (IBM). Jitterplot drawing and correlation analysis was performed using RStudio v. 3.6.3 (https://www.rstudio.com/).

## CONFLICT OF INTEREST

The authors declare that they have no conflict of interest.

## AUTHOR CONTRIBUTIONS

R.T., J.K. and N.C.B. planned and designed the research. R.T. performed experiments, conducted field work, analysed and interpreted data. R.T. and N.C.B. wrote the manuscript and all authors edited and approved the article.

## Supporting information

**FIGURE S1** Amino acid sequence alignment of XLGs of *Arabidopsis thaliana* and *Brassica juncea*

**FIGURE S2** Expression of *Arabidopsis* G‐protein genes in different developmental stages

**FIGURE S3** Multiple sequence alignment of RNAi target regions of *XLG1*, *XLG2* and *XLG3* homologs of *Brassica juncea*

**FIGURE S4** Expression of defence marker genes in *Brassica juncea* XLG‐RNAi lines

**TABLE S1** Pairwise sequence identity (%) between *Brassica juncea XLG* genes and its progenitors with *Arabidopsis XLG* genes

**TABLE S2** Summary of syntenic G‐protein genes identified in the triplicated subgenomes of *Brassica rapa*

**TABLE S3** Pairwise sequence identity (%) calculated among the RNAi target regions of *Brassica juncea XLG* genes

**TABLE S4** Segregation analysis of the RNAi transgenic events of BjuXLG1, BjuXLG2, and BjuXLG3 constructs

**TABLE S5** List of primers used in current study

**TABLE S6** Glucosinolate content in leaf of BjuXLG‐RNAi lines after SSD1 infection

## Data Availability

The data that support the findings of this study are available on request from the corresponding author.
