# Correction to Extra‐Large G‐Proteins Influence Plant Response to *Sclerotinia sclerotiorum* by Regulating Glucosinolate Metabolism in *Brassica juncea*

**DOI:** 10.1111/mpp.70183

**Published:** 2025-12-01

Tiwari, R., J. Kaur, and N. C. Bisht. 2021. “Extra‐Large G‐Proteins Influence Plant Response to *Sclerotinia sclerotiorum* by Regulating Glucosinolate Metabolism in *Brassica juncea*.” *Molecular Plant Pathology* 22, no. 10: 1180–1194. https://doi.org/10.1111/mpp.13096.

Upon re‐examining the original image files, we identified an inadvertent error in Figure 5a. The trypan blue staining panels labelled as BjuXLG2‐RNAi lines 2#11 and 2#21 (representing two independent lines of the same construct) were incorrectly represented, as both panels corresponded to line 2#21. This error occurred during final figure assembly due to the similarity in their file names.

**FIGURE 5:**
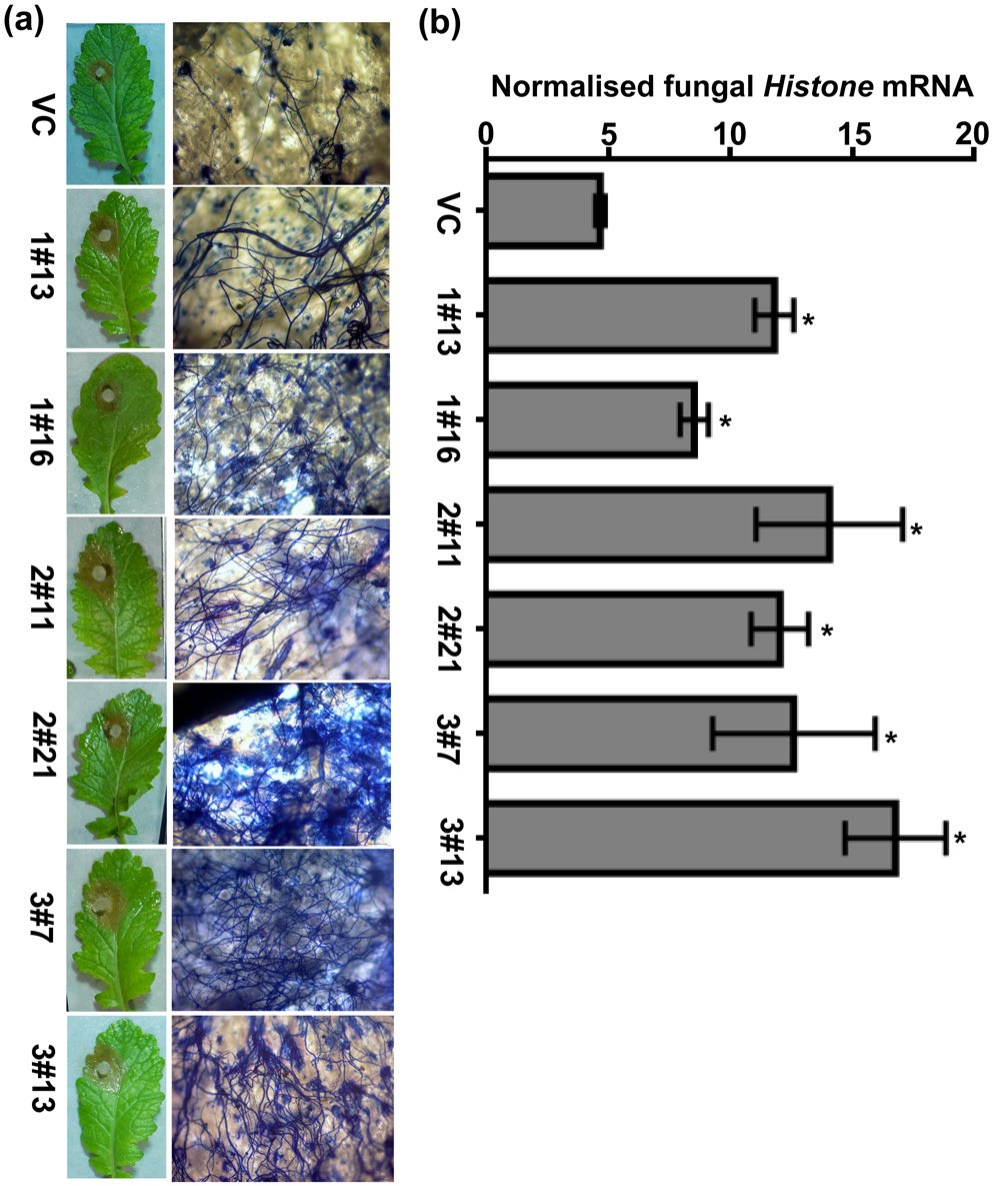
The assessment of *Sclerotinia sclerotiorum* (SSD1) fungal infection in *Brassica juncea* XLG‐RNAi lines. (a) The growth of *S. sclerotiorum* examined by trypan blue staining. Photographs were taken 24‐h post‐inoculation (hpi) and mycelial growth on stained leaves was visualised with bright‐field microscopy. Bar indicates 20 μm. (b) Expression of *S. sclerotiorum* growth on BjuXLG‐RNAi and vector control (VC) plants after SSD1 infection (24 hpi) using reverse transcription‐quantitative PCR. The expression of *S. sclerotiorum* *Histone* mRNA was normalised against the constitutive *BjuTIP41* gene expression level. Three independent experiments were performed, each with two technical repeats. Error bars represent ± *SEM*. Asterisks (*) indicate significant differences (*p* < 0.05, Student's *t* test) between VC and BjuXLG‐RNAi lines.

We have now retrieved and incorporated the correct image corresponding to BjuXLG2‐RNAi line 2#11. The trypan blue staining pattern in the corrected Figure 5a (shown here) remains consistent with the original figure. Specifically, BjuXLG2‐RNAi lines (2#11 and 2#21) infected with *S. sclerotiorum* strain SSD1 exhibit intense blue staining and dense mycelial growth compared to the vector control (VC) plant. This correction does not alter any results, statistical analyses or the overall conclusions of the study.

We sincerely apologise for this error.

