# Supplementary material for: Extra‐large G‐proteins influence plant response to Sclerotinia sclerotiorum by regulating glucosinolate metabolism in Brassica juncea
Source: Mol Plant Pathol. 2021 Aug 10;22(10):1180–94. doi: 10.1111/mpp.13096 (PMC8435238; doi:10.1111/mpp.13096)
Supplement: Supplementary file 1 — FIGURE S1 Amino acid sequence alignment of XLGs of Arabidopsis thaliana and Brassica juncea [file MPP-22-1180-s003.docx]

**
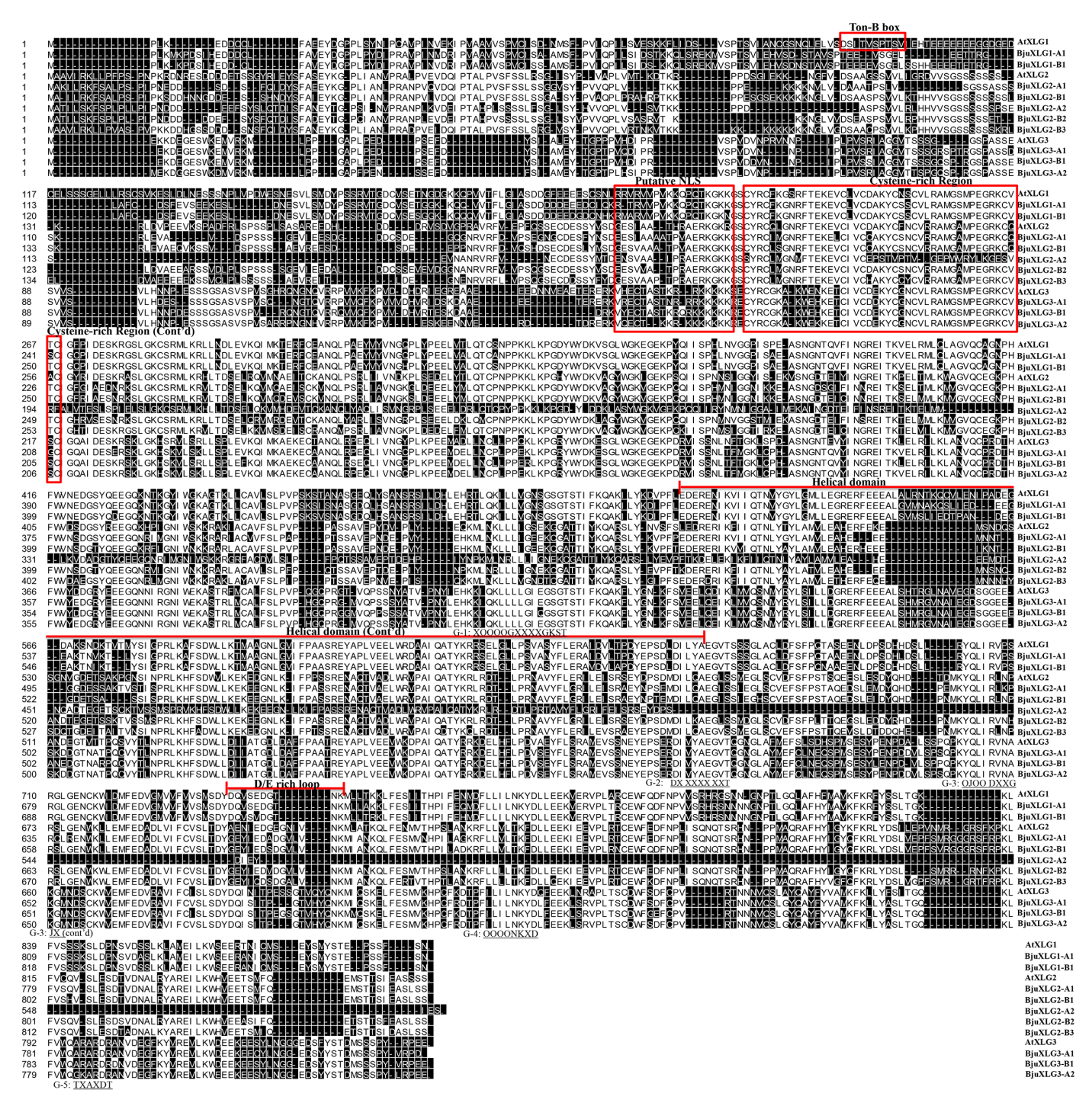
**

**Figure S1: Amino acid sequence alignment of XLGs of *A. thaliana* and *B. juncea*.** The alignment was performed was peformed with MegAlign Tool of DNA star using CLUSTAL V method. Different structural domains of XLGs and consensus sequences of the GTPase domain are marked. The consensus sequences of the GTPase domain are based on Ding et al., 2008. D, aspartic acid; G, glycine; K, lysine; J, hydrophilic residues; N, asparagine; O, hydrophobic residues; S, serine; T, threonine; X, any amino acid is accepted.
