# Supplementary material for: Extra‐large G‐proteins influence plant response to Sclerotinia sclerotiorum by regulating glucosinolate metabolism in Brassica juncea
Source: Mol Plant Pathol. 2021 Aug 10;22(10):1180–94. doi: 10.1111/mpp.13096 (PMC8435238; doi:10.1111/mpp.13096)
Supplement: Supplementary file 2 — FIGURE S2 Expression of Arabidopsis G‐protein genes in different developmental stages [file MPP-22-1180-s008.docx]

**
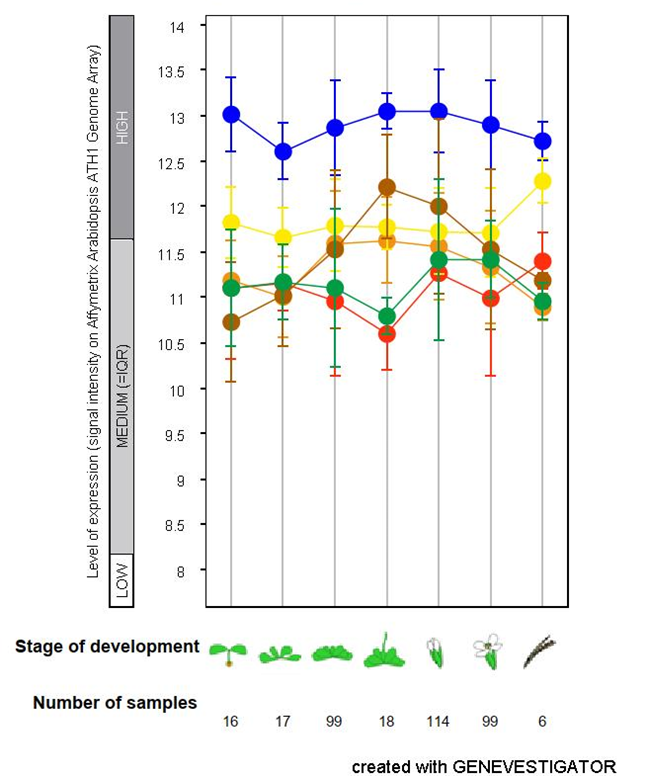
**

**Figure S2:** **Expression of *Arabidopsis* G-protein genes in different developmental stages**. Data of G-protein gene expression during *Arabidopsis* developmental stages (along with number of samples) were obtained from AT_AFFY_AtH1-0 microarray plate. Genes are represented in colored circles- *GPA1* (red), *AGB1* (blue), *AGG1* (orange), *AtXLG1* (yellow)*, AtXLG2* (brown) and *AtXLG3* (green), created in Genevestigator V3. The probes of *AGG2* and *AGG3* were not listed in microarray plate.
