# Supplementary material for: Extra‐large G‐proteins influence plant response to Sclerotinia sclerotiorum by regulating glucosinolate metabolism in Brassica juncea
Source: Mol Plant Pathol. 2021 Aug 10;22(10):1180–94. doi: 10.1111/mpp.13096 (PMC8435238; doi:10.1111/mpp.13096)
Supplement: Supplementary file 3 — FIGURE S3 Multiple sequence alignment of RNAi target regions of XLG1, XLG2 and XLG3 homologs of Brassica juncea [file MPP-22-1180-s006.docx]

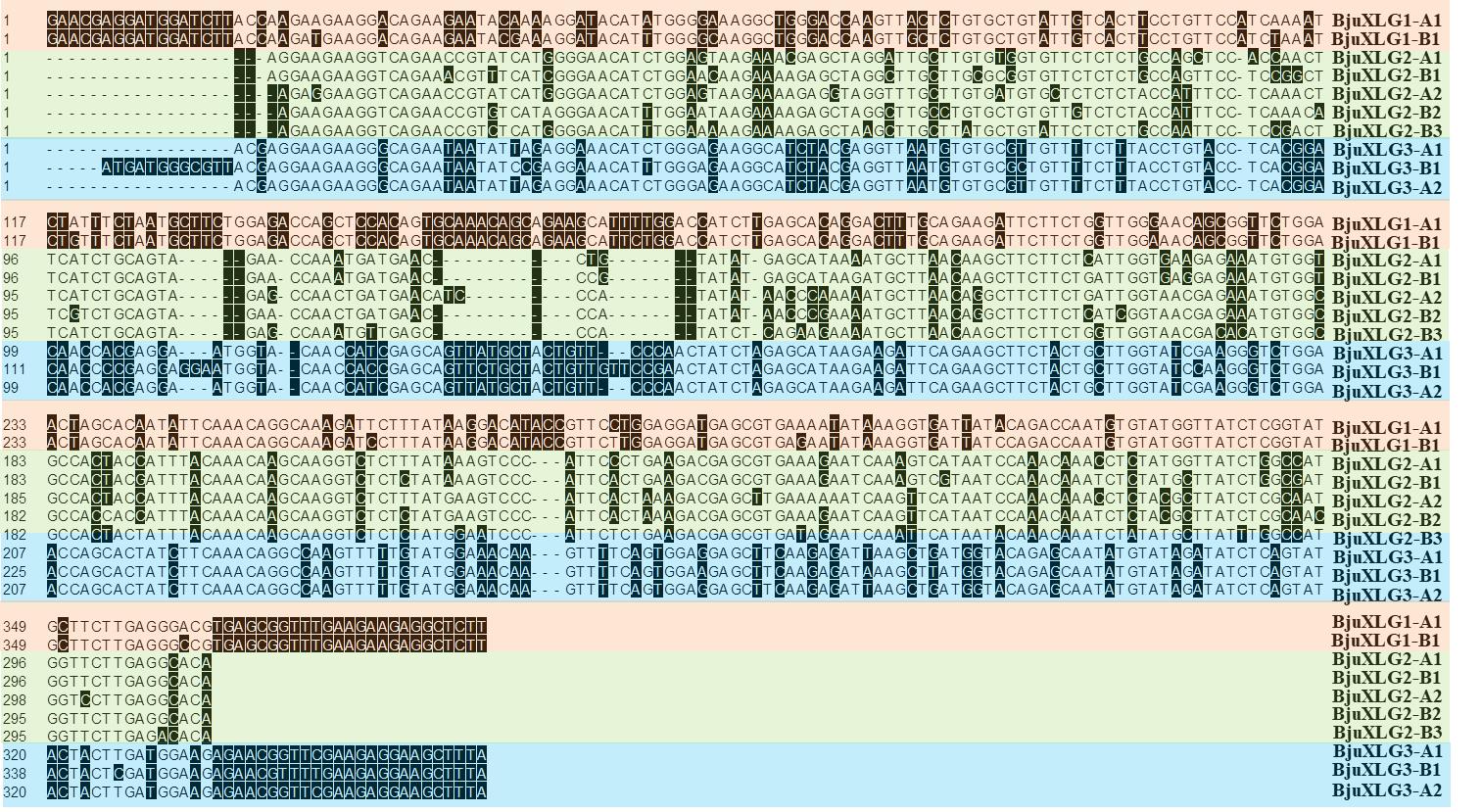


**Figure S3:** **Multiple sequence alignment of RNAi target region of *XLG1*, *XLG2* and *XLG3* homologs of *B. juncea***. Sequence alignment was performed with MegAlign tool of DNASTAR using ClustalW. The background colors for *BjuXLG1* (orange), *BjuXLG2* (green) and *BjuXLG3* (blue) target regions are depicted.
