# Supplementary material for: Extra‐large G‐proteins influence plant response to Sclerotinia sclerotiorum by regulating glucosinolate metabolism in Brassica juncea
Source: Mol Plant Pathol. 2021 Aug 10;22(10):1180–94. doi: 10.1111/mpp.13096 (PMC8435238; doi:10.1111/mpp.13096)
Supplement: Supplementary file 4 — FIGURE S4 Expression of defence marker genes in Brassica juncea XLG‐RNAi lines [file MPP-22-1180-s002.docx]

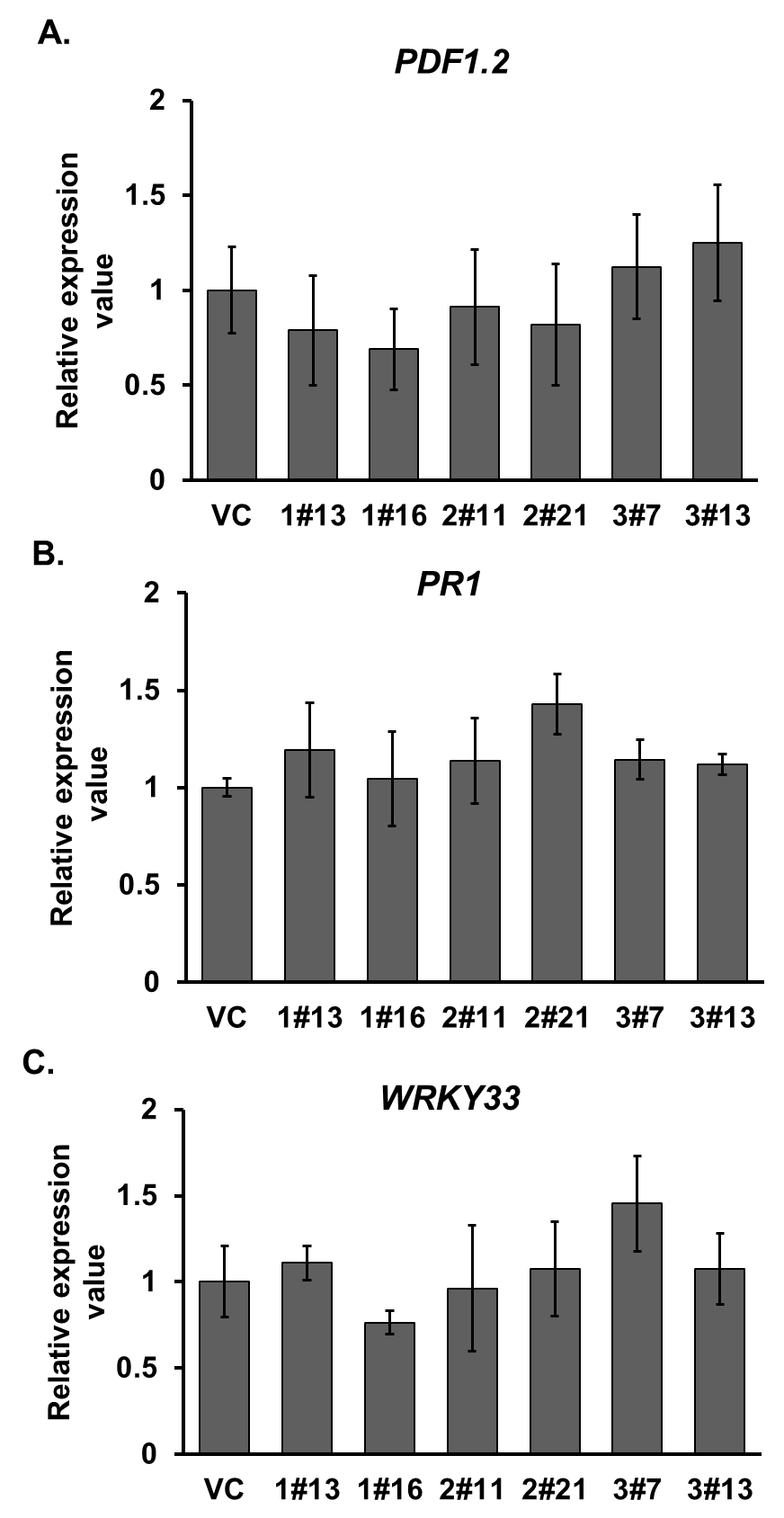


**Figure S4: Expression of defense marker genes in *B. juncea* XLG-RNAi lines.** Fold expression value of defence marker genes **(A)** *PDF1.2* **(B)** *PR1* and **(C)** *WRKY33* in *B. juncea* XLG-RNAi lines and vector control (VC) plants in the non-treated mock samples. Transcripts levels were normalized against the constitutive *TIP41* gene expression level (vector control mock set at 1). Three independent experiments were performed each with two technical repeats and error bars represent +SE of the mean value. No significant differences were observed between VC and BjuXLG-RNAi lines.
