# Supplementary material for: Extra‐large G‐proteins influence plant response to Sclerotinia sclerotiorum by regulating glucosinolate metabolism in Brassica juncea
Source: Mol Plant Pathol. 2021 Aug 10;22(10):1180–94. doi: 10.1111/mpp.13096 (PMC8435238; doi:10.1111/mpp.13096)
Supplement: Supplementary file 5 — TABLE S1 Pairwise sequence identity (%) between Brassica juncea XLG genes and its progenitors with Arabidopsis XLG genes [file MPP-22-1180-s009.docx]

**Table S1**: **Pairwise sequence identity (%) calculated between all shared *B. juncea XLG* genes and its progenitors with *Arabidopsis XLG* genes**. The sequence identity of CDS and amino acid are given in upper right and lower left triangle, respectively. Sequence alignment was performed with MegAlign Tool of DNASTAR using ClustalW.

|  | **(1)** | **(2)** | **(3)** | **(4)** | **(5)** | **(6)** | **(7)** | **(8)** | **(9)** | **(10)** | **(11)** | **(12)** | **(13)** | **(14)** | **(15)** | **(16)** | **(17)** | **(18)** | **(19)** | **(20)** | **(21)** | **(22)** | **(23)** | **(24)** | **(25)** |
| --- | --- | --- | --- | --- | --- | --- | --- | --- | --- | --- | --- | --- | --- | --- | --- | --- | --- | --- | --- | --- | --- | --- | --- | --- | --- |
| *AtXLG1* **(1)** | ** | 84.1 | 84.0 | 83.9 | 84.1 | 48.5 | 46.6 | 46.2 | 45.7 | 45.6 | 42.9 | 48.5 | 48.6 | 48.4 | 49.2 | 49.4 | 48.0 | 50.9 | 51.4 | 51.4 | 51.3 | 51.6 | 50.9 | 51.0 | 50.4 |
| *BraXLG1-A1* **(2)** | 85.9 | ** | 98.2 | 91.5 | 91.4 | 45.4 | 42 | 41.6 | 42.5 | 41.1 | 36 | 35.6 | 41.2 | 41.4 | 39.5 | 43.3 | 42.8 | 48.7 | 43.4 | 43.2 | 41.3 | 41 | 40.1 | 43.7 | 42 |
| *BjuXLG1-A1* **(3)** | 85.8 | 99.4 | ** | 91.4 | 91.1 | 45.4 | 42.3 | 41.5 | 41.7 | 41.2 | 36.4 | 38.2 | 41.6 | 41.5 | 39.5 | 43.2 | 42.8 | 48.8 | 43.4 | 43.3 | 41.4 | 41 | 40.1 | 43.8 | 41.8 |
| *BniXLG1-B1* **(4)** | 84.7 | 93.5 | 93.5 | ** | 98.3 | 46.2 | 39.7 | 41.1 | 41.7 | 41.9 | 36.6 | 37.6 | 39.6 | 40 | 40.3 | 43.9 | 43.3 | 49.3 | 43.3 | 42.5 | 41.1 | 41.7 | 41.6 | 43.4 | 41.8 |
| *BjuXLG1-B1* **(5)** | 84.0 | 93.0 | 93.0 | 99.0 | ** | 46.4 | 41.1 | 40.9 | 41.4 | 41.5 | 36.3 | 37.7 | 39.2 | 41.7 | 39.7 | 43.6 | 42.7 | 49.2 | 42.9 | 42.1 | 40.9 | 41.1 | 41.4 | 43.1 | 41.7 |
| *AtXLG2* **(6)** | 45.4 | 44.1 | 44.2 | 44.5 | 44.4 | ** | 82.7 | 82.2 | 83.4 | 83.0 | 80.5 | 80.3 | 82.7 | 82.5 | 81.7 | 82.6 | 82.2 | 44.6 | 45.6 | 45.6 | 44.8 | 44.9 | 44.7 | 45.1 | 43.6 |
| *BraXLG2-A1* **(7)** | 47.5 | 47.5 | 47.4 | 47.2 | 47.2 | 76.8 | ** | 98.1 | 84.8 | 83.6 | 59 | 63.4 | 79.3 | 75.6 | 72.5 | 77.1 | 77 | 45.9 | 34.1 | 33.9 | 34.2 | 34.2 | 35.7 | 36.8 | 33.7 |
| *BjuXLG2-A1* **(8)** | 47.2 | 46.9 | 46.7 | 47.5 | 47.0 | 75.9 | 99.1 | ** | 84.1 | 83.1 | 58.6 | 62.7 | 73.6 | 73.7 | 73.4 | 77.1 | 76.6 | 45.8 | 35.8 | 36.1 | 33.3 | 32.8 | 35.8 | 34.6 | 31.6 |
| *BniXLG2-B1* **(9)** | 47.7 | 47.0 | 47.0 | 46.7 | 46.5 | 74.9 | 86.6 | 86.4 | ** | 93.2 | 58.5 | 64.6 | 77.7 | 75.8 | 76.7 | 79 | 78.3 | 45.4 | 35.2 | 35.2 | 34.1 | 33.4 | 33.4 | 34 | 35.4 |
| *BjuXLG2-B1* **(10)** | 46.8 | 46.6 | 46.6 | 46.4 | 46.3 | 75.0 | 87.3 | 87.7 | 94.0 | ** | 59.4 | 65.2 | 76.6 | 76.9 | 72.3 | 77 | 80.6 | 40.7 | 35.3 | 35 | 33.3 | 33.3 | 33.1 | 34.4 | 35.4 |
| *BraXLG2-A2* **(11)** | 44.7 | 44.9 | 45.2 | 45.8 | 45.4 | 64.1 | 70.5 | 68.6 | 70.0 | 69.0 | ** | 98.2 | 75.9 | 75.5 | 58.6 | 58.9 | 64.4 | 36.4 | 31.9 | 33.5 | 31.1 | 31.2 | 32 | 31.9 | 32.2 |
| *BjuXLG2-A2* **(12)** | 41.6 | 41.4 | 41.7 | 42.5 | 42.6 | 61.0 | 66.4 | 64.2 | 65.5 | 65.5 | 96.6 | ** | 81.5 | 80 | 62.9 | 63.6 | 64.1 | 45.6 | 34.3 | 34.1 | 34.7 | 34.1 | 34.9 | 33.9 | 34.4 |
| *BniXLG2-B2* **(13)** | 47.1 | 46.3 | 46.5 | 45.8 | 47.0 | 73.6 | 77.1 | 77.6 | 77.9 | 76.7 | 84.2 | 79.5 | ** | 97.9 | 76.1 | 78.4 | 76.5 | 45.7 | 33.2 | 32.9 | 32.9 | 32.8 | 33.6 | 31.3 | 34.9 |
| *BjuXLG2-B2* **(14)** | 47.2 | 45.9 | 46.1 | 46.0 | 46.0 | 73.7 | 78.1 | 77.5 | 76.7 | 76.7 | 83.7 | 78.4 | 98.7 | ** | 75.7 | 77.8 | 76.6 | 45.2 | 32.9 | 33.7 | 33.2 | 33.1 | 32.3 | 31.5 | 33.3 |
| *BraXLG2-A3* **(15)** | 46.6 | 46.9 | 46.8 | 45.5 | 45.3 | 73.7 | 76.6 | 76.4 | 75.2 | 76.5 | 67.3 | 64.4 | 75.3 | 75.4 | ** | 87.1 | 87.4 | 46.7 | 31.3 | 31.4 | 34.5 | 31.9 | 32.9 | 31.4 | 32.5 |
| *BniXLG2-B3* **(16)** | 45.3 | 45.9 | 45.9 | 45.7 | 45.8 | 71.8 | 78.1 | 77.0 | 77.6 | 77.6 | 68.3 | 63.9 | 75.9 | 75.5 | 85.5 | ** | 98.4 | 45.0 | 35.4 | 33.9 | 33.7 | 33.8 | 35 | 33.7 | 33.8 |
| *BjuXLG2-B3* **(17)** | 45.8 | 45.5 | 45.5 | 45.6 | 45.6 | 72.2 | 78.3 | 76.8 | 77.1 | 77.1 | 66.0 | 61.9 | 75.8 | 75.4 | 86.0 | 98.0 | ** | 45.0 | 33 | 33.7 | 34 | 33.7 | 34 | 33.6 | 33.5 |
| *AtXLG3* **(18)** | 44.1 | 43.9 | 44.2 | 44.5 | 44.5 | 38.8 | 38.6 | 38.1 | 38.6 | 38.3 | 37.6 | 35.4 | 37.7 | 37.9 | 38.7 | 38.9 | 38.6 | ** | 87.0 | 87.0 | 87.4 | 86.7 | 86.7 | 87.2 | 87.0 |
| *BraXLG3-A1* **(19)** | 44.9 | 45.2 | 45.2 | 44.9 | 45.3 | 39.4 | 39.6 | 38.9 | 38.6 | 37.9 | 38.2 | 33.9 | 36.8 | 37.8 | 38.2 | 39.2 | 38.2 | 88.9 | ** | 98.7 | 89.6 | 89.4 | 84.9 | 92.6 | 86 |
| *BjuXLG3-A1* **(20)** | 44.0 | 45.5 | 45.5 | 46.2 | 46.0 | 40.3 | 39.0 | 38.6 | 38.9 | 37.8 | 37.6 | 34.5 | 37.3 | 38.1 | 38.2 | 39.5 | 38.4 | 88.9 | 98.3 | ** | 89.5 | 90.3 | 84.8 | 92.2 | 86 |
| *BraXLG3-A2* **(21)** | 45.1 | 44.7 | 44.9 | 45.6 | 44.3 | 39.3 | 39.7 | 38.4 | 38.1 | 37.8 | 38.0 | 35.6 | 38.4 | 38.7 | 38.4 | 39.0 | 37.8 | 88.3 | 86.3 | 87.3 | ** | 99.2 | 84.9 | 87.2 | 86.6 |
| *BjuXLG3-A2* **(22)** | 46.2 | 45.9 | 45.9 | 46.7 | 45.8 | 39.0 | 39.3 | 34.8 | 38.2 | 37.7 | 37.6 | 34.8 | 37.8 | 38.3 | 38.4 | 39.2 | 38.9 | 87.7 | 91.4 | 91.6 | 94.4 | ** | 85.3 | 87.4 | 82.8 |
| *BniXLG3-B1* **(23)** | 44.4 | 45.3 | 45.5 | 45.5 | 44.8 | 38.8 | 38.8 | 38.4 | 37.9 | 38.3 | 38.0 | 35.2 | 37.3 | 37.1 | 37.7 | 38.5 | 38.3 | 88.5 | 93.0 | 92.7 | 88.9 | 90.2 | ** | 92.8 | 94.3 |
| *BjuXLG3-B1* **(24)** | 43.7 | 45.4 | 45.5 | 45.5 | 45.1 | 38.2 | 39.0 | 38.9 | 38.5 | 38.9 | 37.5 | 34.8 | 36.5 | 37.5 | 38.0 | 38.8 | 38.6 | 88.8 | 92.4 | 92.6 | 87.6 | 89.4 | 99.3 | ** | 90.2 |
| *BniXLG3-B2* **(25)** | 44.4 | 44.1 | 44.2 | 43.9 | 43.6 | 38.8 | 38.2 | 36.9 | 38.0 | 37.8 | 38.0 | 35.9 | 37.5 | 37.7 | 37.5 | 38.3 | 37.4 | 88.2 | 87.7 | 87.6 | 96.7 | 92.2 | 89.2 | 87.9 | ** |
