# Supplementary material for: Extra‐large G‐proteins influence plant response to Sclerotinia sclerotiorum by regulating glucosinolate metabolism in Brassica juncea
Source: Mol Plant Pathol. 2021 Aug 10;22(10):1180–94. doi: 10.1111/mpp.13096 (PMC8435238; doi:10.1111/mpp.13096)
Supplement: Supplementary file 6 — TABLE S2 Summary of syntenic G‐protein genes identified in the triplicated subgenomes of Brassica rapa [file MPP-22-1180-s007.docx]

**Table S2: Summary of syntenic G-protein genes identified in the triplicated sub-genomes of *B. rapa* available in BRAD database (**<http://www.brassicadb.org/>**).**

| **G-protein gene** | **Arabidopsis Locus ID (gene code)** | **Least gene fractionized (LF)** | **Moderately gene fractionized (MF1)** | **Most gene fractionized (MF2)** |
| --- | --- | --- | --- | --- |
| XLG1 | At2g23460 (AtXLG1) | - | Bra032166 | - |
| XLG2 | At4g34390 (AtXLG2) | Bra011526 | Bra017647 | Bra034623 |
| XLG3 | At1g31930 (AtXLG3) | Bra023220 | - | Bra033865 |
