# Supplementary material for: Extra‐large G‐proteins influence plant response to Sclerotinia sclerotiorum by regulating glucosinolate metabolism in Brassica juncea
Source: Mol Plant Pathol. 2021 Aug 10;22(10):1180–94. doi: 10.1111/mpp.13096 (PMC8435238; doi:10.1111/mpp.13096)
Supplement: Supplementary file 7 — TABLE S3 Pairwise sequence identity (%) calculated among the RNAi target regions of Brassica juncea XLG genes [file MPP-22-1180-s005.docx]

**Table S3:** **Pairwise sequence identity (%) calculated among the RNAi target regions of *B. juncea XLG* genes**. Sequence alignment was performed with MegAlign Tool of DNASTAR using ClustalW.

|  | **(2)** | **(3)** | **(4)** | **(5)** | **(6)** | **(7)** | **(8)** | **(9)** | **(10)** |
| --- | --- | --- | --- | --- | --- | --- | --- | --- | --- |
| ***BjuXLG1-A1* (1)** | 96.4 | 49.0 | 64.7 | 39.0 | 63.4 | 64.7 | 68.0 | 68.0 | 66.0 |
| ***BjuXLG1-B1* (2)** |  | 48.1 | 63.5 | 37.7 | 63.1 | 64.4 | 66.3 | 66.3 | 65.5 |
| ***BjuXLG2-A1* (3)** |  |  | 87.4 | 92.3 | 86.1 | 85.1 | 61.0 | 61.0 | 57.1 |
| ***BjuXLG2-A2* (4)** |  |  |  | 85.5 | 92.2 | 84.5 | 59.6 | 59.6 | 58.0 |
| ***BjuXLG2-B1* (5)** |  |  |  |  | 86.7 | 86.7 | 61.3 | 61.3 | 57.1 |
| ***BjuXLG2-B2* (6)** |  |  |  |  |  | 85.4 | 60.2 | 60.2 | 57.9 |
| ***BjuXLG2-B3* (7)** |  |  |  |  |  |  | 61.5 | 61.5 | 59.5 |
| ***BjuXLG3-A1* (8)** |  |  |  |  |  |  |  | 100.0 | 95.8 |
| ***BjuXLG3-A2* (9)** |  |  |  |  |  |  |  |  | 95.8 |
| ***BjuXLG3-B1* (10)** |  |  |  |  |  |  |  |  | * |
