# Supplementary material for: Extra‐large G‐proteins influence plant response to Sclerotinia sclerotiorum by regulating glucosinolate metabolism in Brassica juncea
Source: Mol Plant Pathol. 2021 Aug 10;22(10):1180–94. doi: 10.1111/mpp.13096 (PMC8435238; doi:10.1111/mpp.13096)
Supplement: Supplementary file 8 — TABLE S4 Segregation analysis of the RNAi transgenic events of BjuXLG1, BjuXLG2, and BjuXLG3 constructs [file MPP-22-1180-s004.docx]

**Table S4: Segregation analysis of the RNAi transgenic events of BjuXLG1, BjuXLG2 and BjuXLG3 constructs.** The chi-square (χ2) value was calculated for 3R:1S ratio (for glufosinate resistance), and value <3.84 were considered as a single-copy insertion (p<0.05). Additionally, 50 T1 seeds of each transgenic line were also used for calculating the germination frequency after 3 days of imbibition.

| **Construct code** | **Name of construct** | **Total number of T1 progeny germinated** | **BR** | **BS** | **ꭓ2 value** |
| --- | --- | --- | --- | --- | --- |
| ***B. juncea* XLG1 RNAi lines** | | | | | |
| 1#1 | BjuXLG1 RNAi#1 | 42 | 38 | 4 | 5.37 |
| 1#2 | BjuXLG1 RNAi#2 | 32 | 32 | 0 | 10.67 |
| 1#3 | BjuXLG1 RNAi#3 | 56 | 50 | 6 | 6.10 |
| 1#5 | BjuXLG1 RNAi#5 | 48 | 38 | 10 | 0.44 |
| 1#7 | BjuXLG1 RNAi#7 | 4 | 4 | 0 | 1.33 |
| 1#8 | BjuXLG1 RNAi#8 | 5 | 4 | 1 | 0.07 |
| 1#11 | BjuXLG1 RNAi#11 | 1 | 1 | 0 | 0.33 |
| 1#13 | BjuXLG1 RNAi#13* | 105 | 83 | 22 | 0.92 |
| 1#16 | BjuXLG1 RNAi#16* | 25 | 17 | 8 | 0.65 |
| 1#20 | BjuXLG1 RNAi#20 | 55 | 41 | 14 | 0.01 |
| 1#21 | BjuXLG1 RNAi#21 | 6 | 6 | 0 | 2.00 |
| 1#22 | BjuXLG1 RNAi#22 | 50 | 35 | 15 | 0.67 |
| 1#23 | BjuXLG1 RNAi#23 | 27 | 22 | 5 | 0.60 |
| 1#26 | BjuXLG1 RNAi#26* | 51 | 40 | 11 | 0.32 |
| 1#27 | BjuXLG1 RNAi#27 | 89 | 89 | 0 | 29.67 |
| *B. juncea* XLG2 RNAi lines | | | | | |
| 2#1 | BjuXLG2 RNAi#1 | 30 | 24 | 6 | 0.40 |
| 2#2 | BjuXLG2 RNAi#2 | 47 | 34 | 13 | 0.18 |
| 2#3 | BjuXLG2 RNAi#3* | 96 | 71 | 25 | 0.06 |
| 2#4 | BjuXLG2 RNAi#4 | 70 | 53 | 17 | 0.02 |
| 2#7 | BjuXLG2 RNAi#7 | 44 | 44 | 0 | 14.67 |
| 2#8 | BjuXLG2 RNAi#8 | 77 | 63 | 14 | 1.91 |
| 2#9 | BjuXLG2 RNAi#9 | 54 | 38 | 16 | 0.62 |
| 2#11 | BjuXLG2 RNAi#11* | 74 | 58 | 16 | 0.45 |
| 2#12 | BjuXLG2 RNAi#12 | 81 | 63 | 18 | 0.33 |
| 2#16 | BjuXLG2 RNAi#16 | 7 | 6 | 1 | 0.43 |
| 2#18 | BjuXLG2 RNAi#18 | 44 | 28 | 16 | 3.03 |
| 2#21 | BjuXLG2 RNAi#21* | 47 | 34 | 13 | 0.18 |
| *B. juncea* XLG3 RNAi lines | | | | | |
| 3#1 | BjuXLG3 RNAi#1 | 5 | 4 | 1 | 0.07 |
| 3#2 | BjuXLG3 RNAi#2 | 72 | 72 | 0 | 24.00 |
| 3#3 | BjuXLG3 RNAi#3* | 83 | 64 | 19 | 0.20 |
| 3#6 | BjuXLG3 RNAi#6 | 29 | 23 | 6 | 0.29 |
| 3#7 | BjuXLG3 RNAi#7* | 111 | 75 | 36 | 3.27 |
| 3#8 | BjuXLG3 RNAi#8 | 62 | 42 | 20 | 1.74 |
| 3#9 | BjuXLG3 RNAi#9 | 42 | 34 | 8 | 0.79 |
| 3#10 | BjuXLG3 RNAi#10 | 244 | 172 | 72 | 2.64 |
| 3#13 | BjuXLG3 RNAi#13* | 64 | 50 | 14 | 0.33 |
| 3#14 | BjuXLG3 RNAi#14 | 132 | 106 | 26 | 1.98 |
| 3#17 | BjuXLG3 RNAi#17 | 191 | 148 | 43 | 0.63 |
| 3#19 | BjuXLG3 RNAi#19 | 52 | 39 | 13 | 0.00 |
| 3#20 | BjuXLG3 RNAi#20 | 134 | 117 | 17 | 10.84 |
| 3#21 | BjuXLG3 RNAi#21 | 243 | 178 | 65 | 0.40 |
| 3#22 | BjuXLG3 RNAi#22 | 72 | 57 | 15 | 0.67 |
| 3#26 | BjuXLG3 RNAi#26 | 84 | 66 | 18 | 0.57 |
| 3#34 | BjuXLG3 RNAi#34 | 28 | 18 | 10 | 1.71 |

Lines marked with asterisks (*) were used for detailed analysis following identification of single-copy events and gene expression profile.
