# Supplementary material for: Extra‐large G‐proteins influence plant response to Sclerotinia sclerotiorum by regulating glucosinolate metabolism in Brassica juncea
Source: Mol Plant Pathol. 2021 Aug 10;22(10):1180–94. doi: 10.1111/mpp.13096 (PMC8435238; doi:10.1111/mpp.13096)
Supplement: Supplementary file 9 — TABLE S5 List of primers used in current study [file MPP-22-1180-s010.docx]

**Table S5: List of primers used in current study.**

| **S. No.** | **Primer Code** | **Sequence (5’-3’)** |
| --- | --- | --- |
| Amplification of *Brassica* *XLG* genes | | |
| 1 | Bra032166 FP | CACCATGCCATTGAAGATGAAACCCAG |
| 2 | Bra032166 RP | TCAATTCGAGAAGGAACTTGGCTC |
| 3 | Brara.A0038.1 FP | CACCATGGGAAACAGGTTTACTG |
| 4 | Brara.A0038.1 RP | TCAAGAGGATAAGCTGGCCTCGAT |
| 5 | Brara.A0038.1 mid RP | AACATTTCCAGCAATTTCCAG |
| 6 | Bra017647 FP | CACCATGGCTACTATTCTAAGCAAG |
| 7 | Bra017647 RP | TCAGCTTTCAATATATCCAATATTAG |
| 8 | Bra017647 mid RP | TTCTTACTCCAGATGTTCCC |
| 9 | Bra034623 FP | CACCATGGCTGCTGTTTTAAGA |
| 10 | Bra034623 RP | TCAAGAGGATAAGCTGGCATC |
| 11 | Bra023220 FP | CACCATGGAGAAAGATGAAG |
| 12 | Bra023220 RP | TCACTCGTCAGGTCTAACATAC |
| 13 | Bra033865 FP | CACCATGATGGAGAAAGATG |
| 14 | Bra033865 RP | TCACTCCTCAGGTCTAAGATATG |
| Expression profiling of different *XLG* homologs through qRT-PCR | | |
| 15 | AtXLG1 RT FP | GCAGAAGGAGTCACTTCATCCAG |
| 16 | AtXLG1 RT RP | AACCATTCCGACGTCTTCAAAC |
| 17 | AtXLG2 RT FP | GAACCAGACGAGCAGACACA |
| 18 | AtXLG2 RT RP | CCTCAGTGCATTGTCCACAG |
| 19 | AtXLG3 RT FP | CGTATAGCGGGAGGAGTCAC |
| 20 | AtXLG3 RT RP | CACAACCGGTCTACGAACCT |
| 21 | ATTIP41 RT FP | GTGAAAACTGTTGGAGAGAAGCAA |
| 22 | ATTIP41 RT FP | TCAACTGGATACCCTTTCGCA |
| 23 | BjuXLG1-A1 RT FP | TCCAGAAGAGGACACGTGTC |
| 24 | BjuXLG1-A1 RT RP | TAGGACACCCAATACAACTA |
| 25 | BjuXLG2-A1 RT FP | ACGGGAGGAGATGAGAGCAG |
| 26 | BjuXLG2-A1 RT FP | TGTACGTGGCTTGTATGGCA |
| 27 | BjuXLG2-A2 RT FP | GACCGATGAGAACAGCGTA |
| 28 | BjuXLG2-A2 RT RP | ACTCAGAGACTTTGGC |
| 29 | BjuXLG2-A3 RT FP | AGGTTAAATCCAAGAAGGCA |
| 30 | BjuXLG2-A3 RT RP | ATCTTGTTCACAAGAACACCA |
| 31 | BjuXLG3-A1 RT FP | CGAGGAGAAGCTAAGCCGCGT |
| 32 | BjuXLG3-A1 RT RP | GCTCGGTCCCTAGCCCTAG |
| 33 | BjuXLG3-A2 RT FP | CGAGGAGAAGCTTAACCGTTC |
| 34 | BjuXLG3-A2 RT RP | GCTCTATCCCTAGCCTTAC |
| 35 | BjuXLG3-A2 RT FP2 | CGACCTCTTCGAGGAGAAG |
| 36 | BjuXLG3-A2 RT RP2 | ACACAAACAGTTTCTGGCCC |
| 37 | BjuXLG1-B1 RT FP | ACCACAAGAGGATGGCTCGTGTT |
| 38 | BjuXLG1-B1 RT RP | AGGACAGCCGATACAAGTC |
| 39 | BjuXLG2-B1 RT FP | GTTAAATCCAAGAAGCCTT |
| 40 | BjuXLG2-B1 RT RP | TTCACAAGAACCCCGTCGCTG |
| 41 | BjuXLG2-B2 RT FP | GCCACGATCCTAATATGAAGT |
| 42 | BjuXLG2-B2 RT FP | CCACTCGCATGTTCTTAG |
| 42 | BjuXLG2-B3 RT FP | CGATTAAATCCGAGAAGGCTTG |
| 44 | BjuXLG2-B3 RT RP | ATCTTGTTCACAAGAGCCCCATCACT |
| 45 | BjuXLG3-B1 RT FP | CGAGGAGAAACTGAGCCGTGT |
| 46 | BjuXLG3-B1 RT RP | TCTCTGTCCCTAGCCCTAG |
| 47 | BjuXLG3-B2 RT FP | TGATCTCTTCGAGGAGAAGC |
| 48 | BjuXLG3-B2 RT RP | GCCACACAAACAGTTTCTGACCA |
| 49 | BjuTIP41 RT FP | TGAAGAGCAGATTGATTTGGCT |
| 50 | BjuTIP41 RT RP | ACACTCCATTGTCAGCCAGTT |
| 51 | Bjuactin FP | CTTCTTACCGAGGCTCCTCT |
| 52 | Bjuactin RP | AAGGATCTTCATGAGGTAATCAGT |
| 53 | BjuPR1 RTFP | AAGGGTTCACAACCAGGCAC |
| 54 | BjuPR1 RTRP | CCAGGCTAAGTTTTCCCCGT |
| 55 | BjuPDF1.2 RTFP | TCTCTTCGCTGCTCTCGTTCT |
| 56 | BjuPDF1.2 RTFP | ATCCATGTCGTGCTCCCTCAA |
| 57 | BjuWRKY33 RTFP | GAAAGCGAAACGAACGGTGGA |
| 58 | BjuWRKY3RTFP | TGGTGCTGAAGAAGGATCTTGCG |
| 59 | Ss Histone3 FP RT | GGCTCGTACCAAGCAAACTG |
| 60 | Ss Histone3 RP RT | GAAGTCTTGGGCGATTTCAC |
| Primers for development of RNAi constructs | | |
| 61 | XLG1 con RNAi FP | CACCGAACGAGGATGGATCTTACCAA |
| 62 | XLG1 con RNAi RP | AAGAGCCTCTTCTTCAAACCGCTCA |
| 63 | XLG2 con RNAi FP | CACCAGAAGAAGGTCAGAACCGT |
| 64 | XLG2 con RNAi RP | TGTGCCTCAAGAACCATGGCC |
| 65 | XLG3 con RNAi FP | CACCATGATGGGCGTTACGAGGAAG |
| 66 | XLG3 con RNAi RP | CAAAGCTTCCTCTTCAAACCGTTCT |
