# Supplementary material for: Extra‐large G‐proteins influence plant response to Sclerotinia sclerotiorum by regulating glucosinolate metabolism in Brassica juncea
Source: Mol Plant Pathol. 2021 Aug 10;22(10):1180–94. doi: 10.1111/mpp.13096 (PMC8435238; doi:10.1111/mpp.13096)
Supplement: Supplementary file 10 — TABLE S6 Glucosinolate content in leaf of BjuXLG‐RNAi lines after SSD1 infection [file MPP-22-1180-s001.docx]

**Table S6: Glucosinolate content (µmol/g dry weight) in leaf of BjuXLG-RNAi lines after SSD1 infection**. SSD1 infection assays were performed in 20 day old of T3 homozygous plants, and glucosinolates were estimated using HPLC. Data represent the mean±standard error (n=4). Letters on top indicate values significantly different from each other as determined by one-way ANOVA followed by Tukey's *post‐hoc*test (*P*≤ 0.05). Different letters indicate significant differences among plant genotypes, lower case letters for mock plants and upper-case letters for SSD1 infected plants. Abbreviations: GSL, glucosinolate; 4OHI3M, 4-hydroxyindol-3-ylmethyl; I3M, indol-3-ylmethyl; 4MOI3M, 4-methoxyindol-3-ylmethyl.

| **Lines** | **Allyl** | **3-butenyl** | **4-pentenyl** | **4OHI3M** | **I3M** | **4MOI3M** | **Aliphatic GSL** | **Indolic GSL** | **Total GSL** |
| --- | --- | --- | --- | --- | --- | --- | --- | --- | --- |
| VC_mock | 13.06±0.02 | 81.43±0.04 | 1.23±0.00 | 0.13±0.00 | 0.17±0.00 | 0.08±0.00 | 96.77±0.06^b^ | 0.39±0.00^b^ | 97.16±0.06^b^ |
| VC_3hpi | 15.46±0.86 | 91.15±5.64 | 1.68±0.07 | 0.08±0.04 | 0.18±0.09 | 0.00±0.00 | 108.29±6.43^C^ | 0.26±0.13^A^ | 108.55±6.56^C^ |
| 1#13_mock | 12.70±0.62 | 70.70±0.89 | 1.54±0.20 | 0.12±0.06 | 0.15±0.08 | 0.05±0.03 | 85.13±1.61^a^ | 0.33±0.16^ab^ | 85.46±1.45^a^ |
| 1#13_3hpi | 12.14±0.97 | 64.31±5.00 | 1.28±0.09 | 0.00±0.00 | 0.00±0.00 | 0.00±0.00 | 77.73±6.06^B^ | 0.00±0.00^A^ | 77.73±6.06^B^ |
| 1#16_mock | 12.89±0.05 | 69.51±0.33 | 1.68±0.01 | 0.00±0.00 | 0.00±0.00 | 0.00±0.00 | 84.08±0.28^a^ | 0.40±0.13^a^ | 84.08±0.28^a^ |
| 1#16_3hpi | 11.95±0.77 | 64.80±5.49 | 1.26±0.07 | 0.04±0.04 | 0.15±0.07 | 0.00±0.00 | 78.01±6.34^B^ | 0.19±0.10^A^ | 78.20±6.41^B^ |
| 2#11_mock | 12.85±0.90 | 68.85±2.92 | 1.49±0.11 | 0.09±0.04 | 0.12±0.06 | 0.02±0.02 | 83.55±4.05^a^ | 0.23±0.12^ab^ | 83.79±4.11^a^ |
| 2#11_3hpi | 12.35±0.80 | 77.57±2.41 | 1.63±0.03 | 0.10±0.06 | 0.31±0.03 | 0.02±0.01 | 91.62±3.03^B^ | 0.43±0.02^A^ | 92.06±3.04^B^ |
| 2#21_mock | 11.78±0.22 | 66.21±1.95 | 1.38±0.08 | 0.13±0.05 | 0.31±0.02 | 0.05±0.01 | 79.55±2.27^a^ | 0.50±0.04^ab^ | 80.05±2.28^a^ |
| 2#21_3hpi | 9.72±0.75 | 63.31±3.61 | 1.51±0.04 | 0.09±0.04 | 0.16±0.09 | 0.02±0.01 | 70.06±3.92^AB^ | 0.20±0.07^A^ | 70.26±3.99^AB^ |
| 3#7_mock | 11.46±0.56 | 67.06±3.40 | 1.46±0.11 | 0.00±0.00 | 0.02±0.02 | 0.01±0.01 | 80.63±3.50^a^ | 0.03±0.02^a^ | 80.66±3.50^a^ |
| 3#7_3hpi | 12.11±0.59 | 62.14±3.38 | 0.90±0.45 | 0.09±0.04 | 0.06±0.03 | 0.06±0.03 | 75.15±4.42^B^ | 0.15±0.08^A^ | 75.30±4.50^B^ |
| 3#13_mock | 11.92±0.45 | 70.07±2.24 | 1.48±0.15 | 0.00±0.00 | 0.00±0.00 | 0.00±0.00 | 81.96±2.52^a^ | 0.01±0.00^a^ | 81.97±2.52^a^ |
| 3#13_3hpi | 8.02±0.20 | 47.82±1.69 | 0.85±0.42 | 0.14±0.07 | 0.17±0.09 | 0.02±0.01 | 56.69±2.31^A^ | 0.33±0.17^A^ | 57.02±2.48^A^ |
